# Effectiveness of Elements of Social Behavior Change Activities in Nutrition-Sensitive Agriculture Programs: A Systematic Review

**DOI:** 10.1016/j.cdnut.2024.104420

**Published:** 2024-07-26

**Authors:** Tsedenia Tewodros, Carolina X Escobar, Liris S Berra, Amy Webb Girard

**Affiliations:** 1 Hubert Department of Global Health, Rollins School of Public Health at Emory University, Atlanta, GA, United States; 2 Nutrition and Health Sciences, Emory University, Atlanta, GA, United States

**Keywords:** nutrition, agriculture, social behavior change, diet diversity, project implementation

## Abstract

**Background:**

Limited research exists on the specific approaches and behavior change techniques (BCT) used in nutrition-sensitive agriculture (NSA) programs and their effects on diet diversity.

**Objectives:**

We aimed to describe nutrition-related social behavior change (SBC) in the context of NSA and quantify the effectiveness of different SBC components of NSA programs in improving diet diversity.

**Methods:**

We searched PubMed, Embase, Web of Science, the International Food Policy and Research Institute repository, and Agricola for articles published between 2000 and 2023. We identified the agricultural activities each project used as a pathway to improved nutrition (ag-nutrition pathways), identified SBC approaches used by each project, and coded BCTs using validated coding protocols. Effectiveness ratios (ERs) were calculated to assess pathways, approaches, and BCTs in relation to dietary diversity outcomes (minimum diet diversity for children, child dietary diversity score, and women’s dietary diversity).

**Results:**

Of 65 included NSA interventions, the most used agriculture-to-nutrition pathways included *1*) *agricultural production for home consumption* (*n* = 61); *2*) *women’s empowerment* (*n* = 36); and *3*) *agricultural income* (*n* = 37) pathways. The most used SBC approaches were interpersonal communication (IPC, *n* = 59) and community-based approaches (*n* = 53). Frequently used BCTs included “instructions on how to perform the behavior” (*n* = 65), “social support (unspecified)” (*n* = 43), and using a “credible source” (*n* = 43). The increased production for the home consumption pathway, IPC approach, and the BCT “behavioral practice” had high ERs for diet diversity outcomes.

**Conclusions:**

Although the agricultural production for home consumption pathway to improved nutrition had the highest ERs for diet diversity, other pathways, such as income generation and reducing wastage, hold promise and require additional investigation. The most commonly applied BCTs focused on information dissemination; however, participatory BCTs related to behavioral demonstration, and behavioral practice had higher ERs. Findings indicate a need to test less frequently utilized SBC components to determine effectiveness.

This trial was registered at PROSPERO (=https://www.crd.york.ac.uk/prospero/display_record.php?RecordID=179016) as CRD42020179016.

## Introduction

Nutrition-sensitive agriculture (NSA) utilizes agricultural strategies to improve household and individual diet quality and nutrition by addressing underlying determinants of suboptimal intakes of nutritious foods [1]. Herforth and Harris [2] outline 3 *primary and interrelated* pathways through which agricultural activities can improve nutritional outcomes:

1. Food production – community members with access to farmland produce more diverse and nutritious foods; availability for year-round household consumption may be increased through enhanced production, processing, and storage practices.
2. Agricultural income – increased household income through agricultural activities can be used for both nutritious food and nonfood purchases that lead to improved nutrition and health.
3. Women’s empowerment – increased income and decision-making power for women, especially in rural populations, increases spending on nutritious food, and improves nutritional status for women and children. Time and labor-saving efforts can reduce the impacts of women’s agricultural work on their own and their children’s health and nutrition.

Duncan et al. [3] describe similar pathways from agricultural activities to improved nutrition but with additional emphasis on decreased food prices and the role of women’s employment, demands on women’s time and energy expenditure, and health status, indeed identifying these as additional separate pathways. A more detailed description of agriculture-to-nutrition pathways is found in Supplemental Appendix A.

Research consistently demonstrates NSA’s ability to significantly improve both women's and children’s diets in low- and middle-income countries (LMIC), notably through increased consumption of high-quality proteins, vitamin A-rich fruits and vegetables, and enhanced diet diversity in general [[4], [5], [6], [7], [8], [9]]. Early research in the field of NSA observed that activities focused on changing diet behaviors were indispensable components of agriculture programs seeking to enhance nutrition and improve diet quality [10]. These activities range, for example, from traditional nutrition education to more interactive cooking demonstrations and recipe contests to social behavior change (SBC) strategies that seek to shift social and cultural food and gender norms. However, NSA programs that include diet-related behavior change activities often demonstrate less than anticipated impacts [6]. Although previous reviews have cited weak evaluation designs and inconsistent use of different outcome and impact metrics, or questioned the potential effectiveness of these programs, it is also plausible that inconsistent findings stem from the heterogeneity across the behavior change activities that target diet behaviors, the appropriateness of the selected behavior change activities and the quality of their implementation [4,6,9]. Indeed, little is known about how diet-related behavior change activities conducted in the context of NSA are designed and implemented. Although long recognized as critical to achieve diet-related impacts, there has been limited systematic review and examination of the behavior change strategies developed and implemented as part of NSA interventions to improve diet outcomes.

Behavior change techniques (BCTs), often referred to as the “active ingredients” of a behavior change intervention, are specific, fundamental actions that can be observed and replicated. Following a Delphi process of behavior change interventions, Michie et al. [11] identified 93 unique BCTs across 16 activity domains. For example, the activity domain of “Goals and Planning” includes specific BCTs related to behavioral goal setting (1.1), problem-solving (1.2), and action planning (1.4). BCTs in the “social support” domain include both emotional (6.2) and practical (6.3) social support while BCTs in the domain “Antecedents” include BCTs related to restructuring the physical (12.1) and social (12.2) environments. Specific BCTs that make up a behavior change intervention can be identified by coding activities according to this taxonomy for each intervention. A full list of the 16 domains and 93 BCTs is available in Michie et al. 2013 [11] and on the BCT taxonomy training website (https://www.bct-taxonomy.com/).

Because behaviors and the interventions designed to change them are often complex, the process of identifying and mapping BCTs within and across behavior-change interventions using a systematic approach provides a standard for comparing causal mechanisms across interventions. It also enables researchers to isolate the effective components of behavior change interventions for purposes of replication, collaboration, and enhanced effectiveness. Identification and mapping of BCTs using the BCT taxonomy developed by Michie et al. [11] has been applied to numerous health care and population-level behavior change interventions, including diet-related behavior change interventions for obesity reduction in North America and Europe [[12], [13], [14], [15]] and infant and young child feeding (IYCF) practices in LMIC [16]. Identification and mapping of BCTs used in NSA would support the identification of effective techniques for adoption and scale-up and highlight less utilized techniques that may hold promise but require further research.

To systematically document the implementation and heterogeneity of behavior change programming used in NSA, our team aimed to achieve the following objectives using qualitative and quantitative methods:

Objective 1: Using existing conceptual frameworks [2,3], identify the commonly used agriculture-to-nutrition pathways in NSA interventions.

Objective 2: Characterize the current landscape of SBC design and implementation in NSA projects including the SBC approaches used in NSA.

Objective 3: Characterize the commonly applied BCTs in NSA interventions.

Objective 4: Evaluate the effectiveness of the different agriculture-to-nutrition pathways, SBC approaches, and BCTs used in NSA for improving dietary diversity outcomes.

## Methods

### Literature search and screening

#### Literature search process

To identify relevant articles, we searched various combinations of terms from 4 domains – specific agricultural strategies, behavior change, nutrition, and NSA (Table 1). We searched these combinations on PubMed, Embase, and Web of Science. Additionally, we searched for relevant gray literature on the International Food Policy and Research Institute repository and Agricola, the United States Department of Agriculture’s national agricultural library, which resulted in nonpeer-reviewed discussion articles, program documents, and reports. Our searches were restricted to articles published between January 2000 and December 2021. An additional updated search for studies conducted between 2022 and 2023 was done in August 2023. The full search strategy for this review can be found in online Supplemental Materials Appendix B. This systematic review was registered with and can be accessed via PROSPERO (ID number: CRD42020179016).

**TABLE 1:** Terms used for literature searches on peer-reviewed and gray-literature databases by domain (additional details can be found in Supplemental Appendix F)

Domain	Search terms
1. Agriculture strategies	
Biofortification	biofortif∗ OR bio-fortif∗ OR harvestplus OR “harvest plus”
Homestead food production	“homestead food production” OR “home garden” OR “home gardening”
Livestock and dairy production	(livestock∗ AND programs) OR (livestock∗ AND production) OR (livestock∗ AND ownership) OR (dairy∗ AND production) OR (dairy∗ AND program)
Agriculture extension	(agricultur∗ AND extension)
Aquaculture and fisheries	Aquaculture OR fisheries or fishpond
Irrigation	Irrigation
Value chains	“value chain” OR value-chain OR cereal OR maize OR barley OR sorghum OR farro OR enset OR cassava OR banana OR bean OR legume OR poultry OR egg OR microlivestock OR pulse
2. Behavior change	(“behavior change” OR “behaviour change” OR “Behavior change communication” OR “behaviour change communication” OR “social behavior change” OR “social behaviour change” OR “social marketing” OR “nutrition education”)
3. Nutrition outcomes	“Nutrition outcome” OR “nutritional outcome” OR “nutrition status” OR “nutritional status” OR “diet diversity” OR “dietary diversity” OR “diet diversification” OR “dietary diversification”
4. Nutrition-sensitive agriculture	(“nutrition-sensitive” OR “nutrition-sensitive”) AND agricultur∗

#### Inclusion and exclusion criteria

Project documents and publications were eligible for inclusion if ≥1 document was published in English and described an intervention or project in an LMIC [17] with clearly defined agricultural activities (see Table 1), nutrition-focused SBC activities, and a goal or objective of improving diet diversity and/or micronutrient adequacy. In the event documents/publications were in a language other than English, we attempted to translate and include as many as possible. It should be noted that we defined nutrition-focused SBC activities as those that targeted increased *consumption* of nutritious foods; therefore, projects that used SBC activities solely targeting the *production* of nutritious foods without activities targeting consumption were excluded. To characterize the SBC approaches used in NSA and mapping BCTs, all experimental research designs were included. Observational studies were excluded.

### *Screening and selection*

**FIGURE 1:**
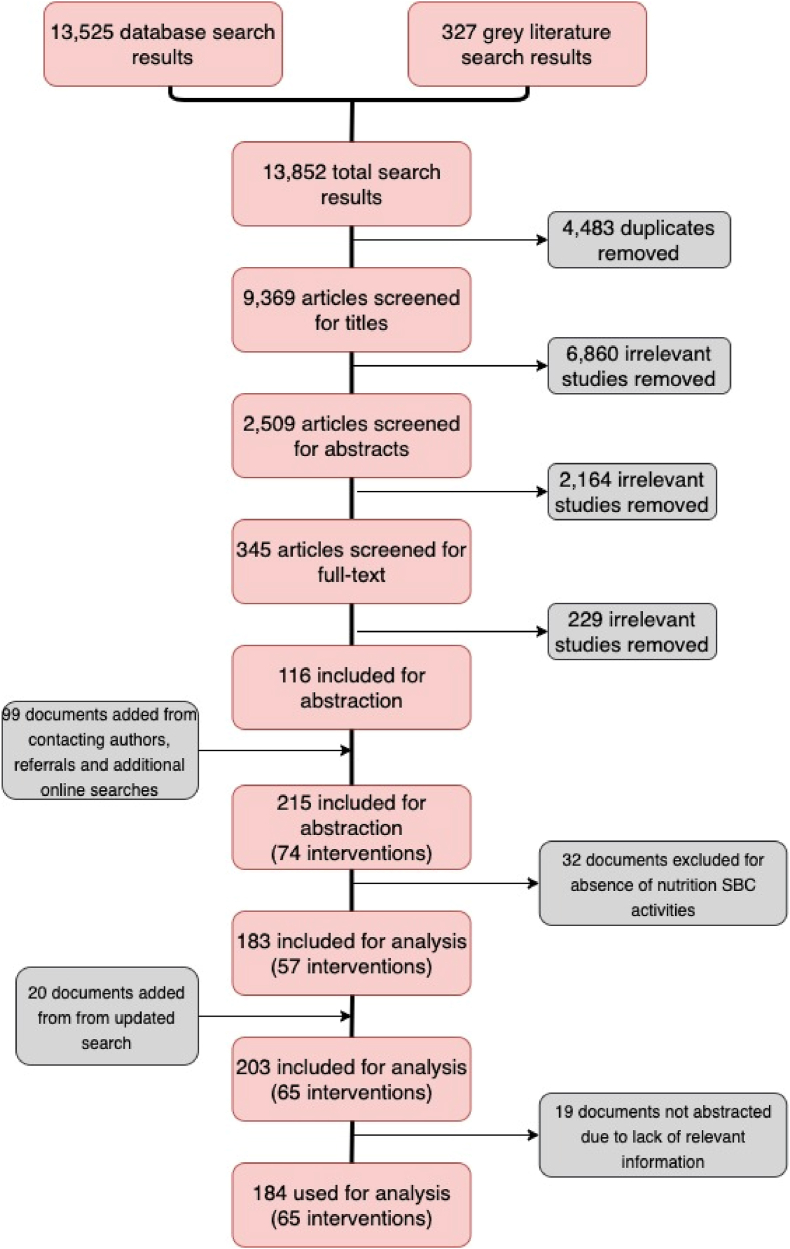
Preferred Reporting Items for Systematic Reviews and Meta-Analyses (PRISMA) flow diagram for studies included in the systematic review.

The database and gray literature searches resulted in 9369 documents after deduplication (Figure 1). Titles, abstracts, and then full texts of these documents were double-screened by co-authors in 3 rounds using Covidence, a systematic review management website (https://www.covidence.org/). At each stage, documents describing interventions that did not meet the inclusion criteria were eliminated. Conflicts in the decision to exclude or include the studies were resolved through discussion between the co-authors.

To increase the depth of information about interventions, the corresponding authors or first authors of included studies were contacted through e-mail and asked to provide additional project documents, such as SBC strategies, formative study reports, evaluations, and nutrition education curricula. We received additional documents from 18 included projects. These additional documents were also used to further ascertain whether the selected interventions met the inclusion criteria and provide greater detail on the SBC activities for purposes of coding BCTs. An additional 20 documents were included after an updated search in 2023.

#### Data abstraction

General *1*) program details, *2*) specific nutrition behavior change activities, and *3*) relevant nutrition outcomes were abstracted from each document into a structured abstraction sheet. Within these 3 broader domains, data about the projects’ formative research results, gender strategy, use of existing government or community systems, target audience, and key messages of behavior change activities, SBC activities, and approaches were also abstracted. Impact estimators for dietary outcomes were abstracted when available and included those related to diet quality, micronutrient adequacy, and consumption of nutritious target foods (for example, orange-fleshed sweet potatoes, eggs, dark green leafy vegetables, vitamin A-rich fruits, and vegetables).

Co-authors initially abstracted the same 3 projects, compared abstraction, and discussed any discrepancies with the principal investigator. Co-authors continued to abstract independently after consistency in the level of data being abstracted was established. Abstractions were reviewed biweekly to ensure agreement and consistency. Where information about a specific project was limited, project websites and other gray literature and peer-reviewed publications on the project were searched for additional information.

### Coding and data analysis

#### Identifying agriculture-to-nutrition pathways

After project documents were fully abstracted, the projects’ intended agriculture-to-nutrition pathways were identified using existing conceptual frameworks [2,3]. Based on what authors reported, we identified whether projects aimed to *1*) increase home production, *2*) increase agricultural income, *3*) increase market availability and accessibility (through increased general production or reduced pricing), *4*) increase women’s empowerment, and *5*) reduce food waste to improve consumption of nutritious foods. A project was categorized as using 1 or more pathways based on project design and implemented activities. For example, if a project provided seeds for cash crops to women’s savings groups, it would be categorized under both the “production for income” and “women’s empowerment” pathways. See Supplemental Appendix A for agriculture-to-nutrition pathways and their descriptions.

#### Identifying SBC approaches used

Supplemental Appendix C, Table C1 lists and defines commonly applied SBC approaches used to influence diet and nutrition-related behaviors. Based on descriptions provided in manuscripts and project documents provided by authors, SBC activities were classified as using 1 or more of the following approaches for purposes of this systematic review: interpersonal communication (IPC), community-based approaches, media, advocacy, entertainment education, social mobilization, positive deviance, social marketing, and behavioral economics. For this study, community-based approaches included community dialog, engagement, mobilization, and outreach. The media category included small media (posters, flyers), mid-media (billboards, community radio), digital/social media, and mass media.

#### Identifying and coding BCTs

We used Taxonomy of BCTs by Michie et al. [11] to identify the BCTs used in each project and qualitatively coded them using MAXQDA (MAX Qualitative Data Analysis) software. To ensure coding consistency, all authors completed online training on BCT taxonomy coding [18], https://www.bct-taxonomy.com/). Documents from the first 5 projects were coded by 3 co-authors to establish inter-coder agreement (85%) and discuss discrepancies in understanding and application of the BCTs.

In addition to those BCTs outlined in the taxonomy, our team created 3 new codes based on commonly occurring techniques in the interventions included in this review. The first was a *promotion* code to capture activities that generally described promoting specific behaviors without providing additional details. For example, a project that says they promoted orange-fleshed sweet potatoes through community health workers or market-based strategies would be coded as a *promotion.* The second was a *role model/social influence* code created to capture activities in which peer social influence was used or an enabling social environment was fostered to make a behavior more acceptable. For example, if a project conducted public graduation ceremonies for mothers who adopted specific nutrition behaviors to make those behaviors desirable to other mothers in the community, it would be coded as a *role model/social influence*. This code differs from the existing social support and behavior comparison codes in the taxonomy because it was used for activities that did not directly address target beneficiaries' behaviors. Instead, the *role model/social influence* code focused on social norms that would have an implicit impact on a person’s willingness to adopt a behavior. The third code we created was a general code titled *behavior maintenance* to capture follow-up activities that supplement the main SBC activity with the explicit aim of supporting continuation of the behavior. For example, this code would be used when participants receive household visits to reinforce behaviors learned during a community-wide event. Online Supplemental Appendix C, Table C2 lists and defines a sample of BCTs and examples from this study.

### Data analysis

#### Calculating effectiveness ratios

We assessed agriculture-to-nutrition pathways, SBC approaches, and BCTs through the estimation of effectiveness ratios (ERs) [12,[19], [20], [21]]. To do this, we first assigned projects as effective or noneffective. Projects that showed a statistically significant positive impact (*P* < 0.05) for the following dietary diversity indicators were classified as effective: *1*) child diet diversity score (DDS-C), *2*) minimum dietary diversity for children (MDD-C), *3*) women’s diet diversity score (DDS-W), and *4*) women’s minimum diet diversity (MDD-W). The majority of projects (*n* = 24 of 28) utilized 24-hour recalls to estimate diet diversity and followed validated guidance for their estimation [[22], [23], [24]]. Three projects used a 7-day recall [[25], [26], [27]], while 1 used a 30-day recall [28]. Due to the relatively lower number of women’s diet diversity indicators, the 2 indicators were analyzed collectively as women’s dietary diversity (WDD).

We then calculated ERs for each of the 3 components (agriculture-to-nutrition pathways, SBC approaches, and BCTs). An ER is defined as the number of times a component was part of an *effective* intervention (numerator) as a ratio of the total number of times that component was used in an intervention (denominator). For example, if a specific BCT was used in 10 interventions that measured MDD-C and 5 were found to be effective in improving MDD-C, the ER for that BCT would be 0.5 or 50%. Similarly, if 5 interventions that measured DDS-C used IPC as an SBC approach and all 5 were found to be effective in improving DDS-C, the ER for IPC would be 1. In addition to each outcome of interest separately, ERs were calculated for the “at least 1 outcome” category. In this case, projects that significantly improved *at least one* of the outcomes of interest were designated as effective. Effectiveness assessments were limited to quasi-experimental or randomized controlled studies that had a counterfactual and included at least baseline and midline or endline data. Twenty-eight of the 65 projects included in this review met these criteria, suggesting potential for reporting bias. The other 37 projects were excluded from ER calculations due to less rigorous evaluation design (that is, absence of a counterfactual, *n* = 13), unavailability of baseline or follow-up evaluation document (*n* = 9), or no dietary diversity outcomes of interest reported (*n* = 15). ERs were only calculated for agriculture-to-nutrition pathways, SBC approaches, and BCTs if used in 4 or more projects to avoid inflation of results; this represents a more conservative estimate compared with previous analyses using the ER approach [12,19,21].

#### Risk of bias assessment and PRISMA reporting

We assessed the risk of bias (RoB) for studies included in ER estimations using an adapted RoB assessment tool drawn from the Cochrane RoB assessment guidelines for cluster randomized controlled trials [29], those outlined by Watson et al. [20] and based on guidance provided by the National Heart, Lung and Blood Institute [30]. Our final RoB assessment included 31 criteria scored as 0 (criteria not met at all), 0.5 (criteria partially met, where applicable), and 1 (criteria fully met). Final bias scores were calculated by summing individual criteria and dividing by 31 with scores closer to 1 indicating less RoB and those closer to 0 indicating greatest RoB. Findings from the RoB assessment by the study are detailed in online Supplemental Appendix D, Tables D1–D3. RoB ratios ranged from 0.48 to 0.94. As expected, interventions applying randomized, controlled evaluation designs had a higher average RoB ratio (0.85) than those applying nonrandomized designs (0.71). Sensitivity analysis was conducted by estimating ERs both retaining and dropping those studies with RoB ratios < 0.70 (*n* = 7). This manuscript was prepared in accordance with relevant PRISMA guidelines [31]; adherence to and deviations from the guidelines are noted in online Supplemental Appendix E. Data abstraction tables and other data synthesis tables are available upon request to the corresponding author.

## Results

Our search was conducted from February to July 2020 and retrieved 9369 unique titles (13,852 before removing duplicates). The title review led to the inclusion of 2509 unique publications for further abstract review, of which 345 were included for full-text review. A full-text review yielded 116 documents on 57 unique interventions to include for abstraction (Figure 1). Contacting authors and conducting additional online searches increased the total number of abstracted documents to 183. A second-round search was conducted in July 2023 and an additional 20 publications were abstracted and included, for a total of 203 documents and 65 unique interventions. A summary of included studies with a complete list of citations can be found in online supplemental materials, Supplemental Appendix F. Examples of studies excluded during abstraction are listed in Supplemental Appendix G.

**FIGURE 2:**
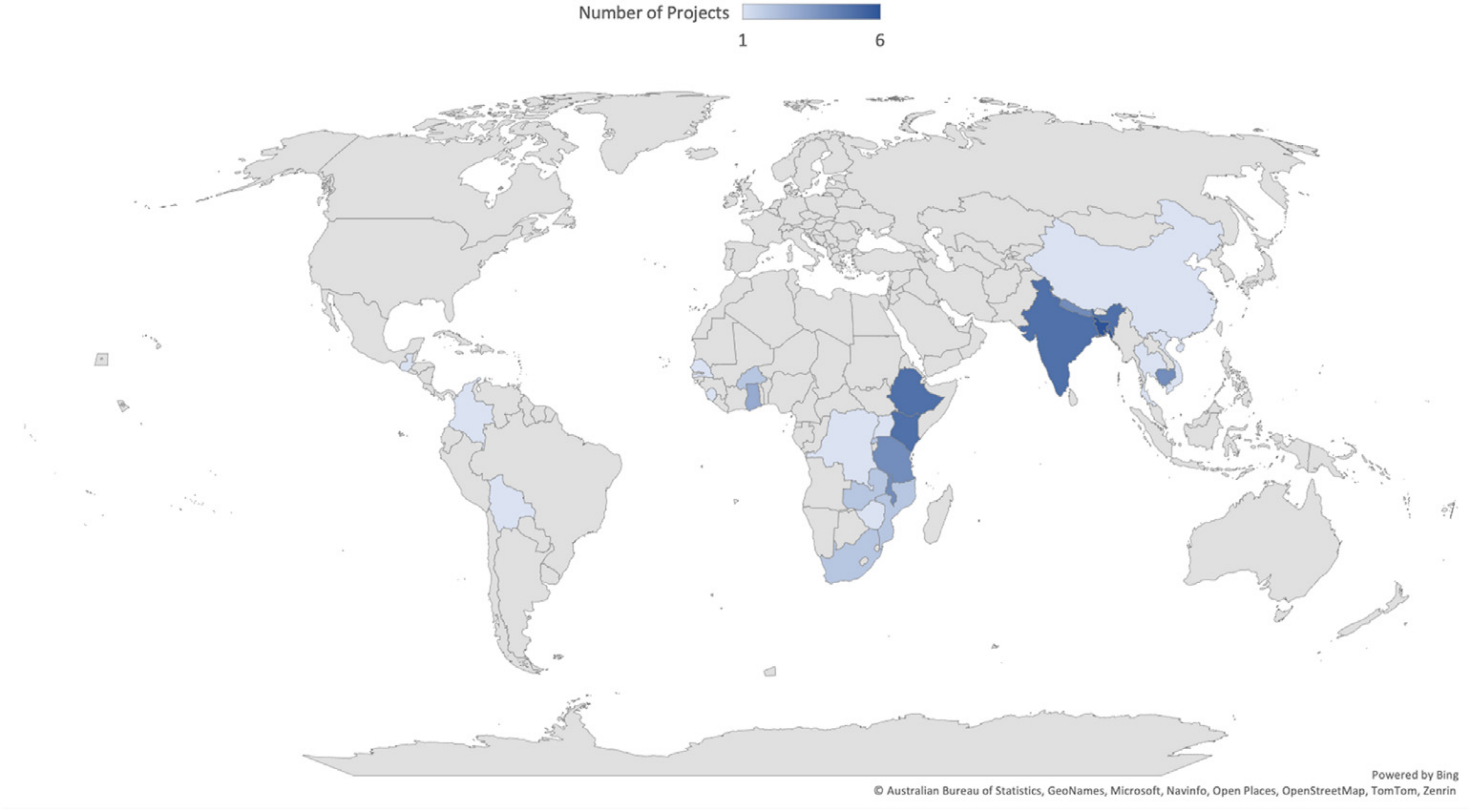
Geographic distribution of the 65 unique interventions included in the systematic review.

Projects were predominantly located in sub-Saharan Africa (*n* = 36) and South Asia (*n* = 16) and World Bank regional classifications (Figure 2). Seven projects were situated in East Asia and the Pacific region and 3 in the Latin America and Caribbean region. Three projects were multi-country interventions, either spanning continents or focusing on countries within the same continent/region. Twenty-three projects targeted improvements in child nutrition, 27 aimed to improve both maternal and child nutrition, and 15 aimed to improve the nutrition status of the entire household. In terms of outcomes measured, of the 38 projects that did not explicitly aim to improve maternal nutrition, 10 measured ≥1 type of maternal nutrition indicator (for example, dietary diversity, nutrient intake, micronutrient status, and food group/item consumption) as an outcome.

Of the 65 projects, only 8 mentioned using specific behavioral theories to guide intervention design and implementation, the most common being the socioecological theory of behavior change (*n* = 4). Thirty-seven projects reported conducting formative research, 27 of which were primary research studies conducted by the project itself. Twelve projects reported utilizing a specific approach or framework to guide intervention design, including participatory research and education (*n* = 5), negotiating for behavior change (*n* = 1), user-centered design (*n* = 1), FAO's integrated agriculture-IYCF nutrition education approach (*n* = 1), BEHAVE Framework for Designing for Behavior Change (DBC, *n* = 2), adult learning approach (*n* = 1), or community-based intervention design approaches (*n* = 1). Thirty projects described and/or presented a diagram of their theory of change or program impact pathway for their project.

### Agriculture-to-nutrition pathways

**FIGURE 3:**
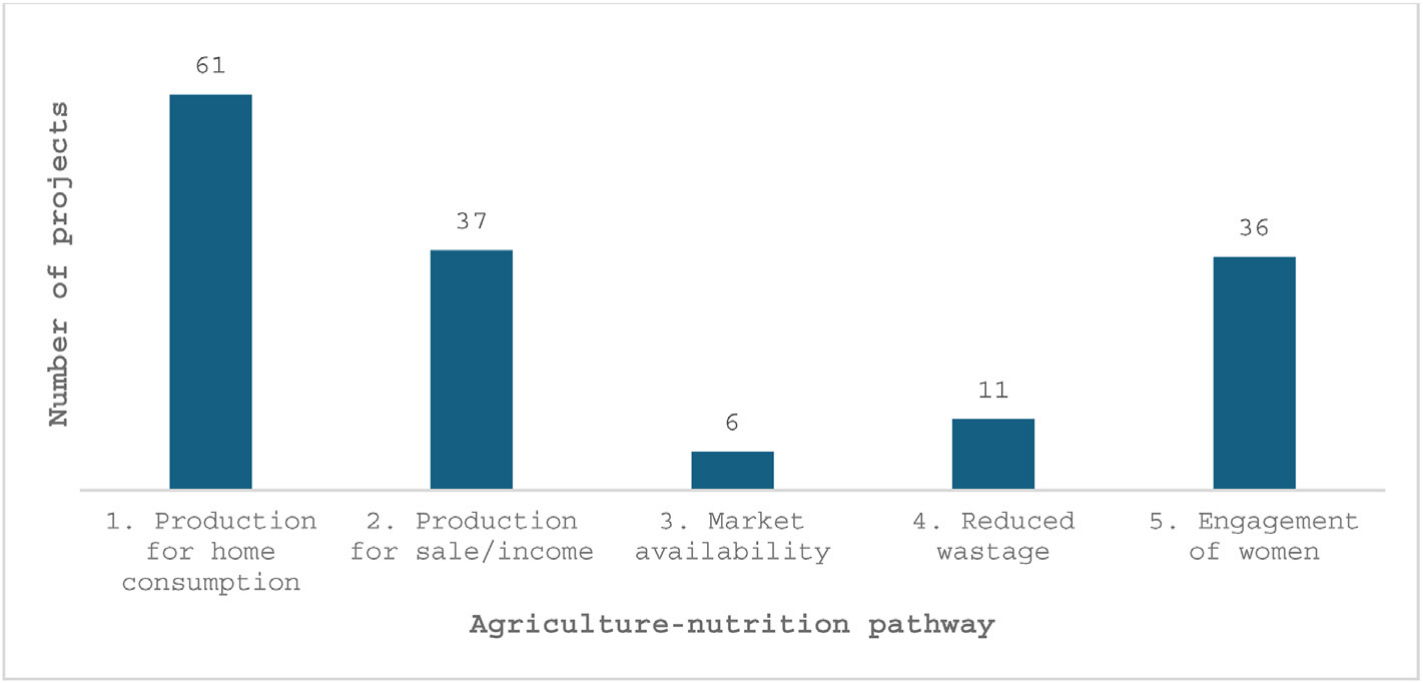
Agriculture-to-nutrition pathways used in the projects included in this review (*n* = 65), based on the agriculture-to-nutrition pathways outlined by Herforth [13]. Projects may have utilized >1 pathway.

The majority of projects implemented activities along the agricultural production for own consumption pathway to improve nutrition outcomes (pathway 1, *n* = 61; Figure 3 [13]). Thirty-three of the projects that emphasized agricultural production for own consumption also reported activities targeting surplus or complementary food production for income generation and use of that income for healthy food or other family-focused expenses (that is, healthcare and sanitation), whereas 4 focused solely on income generation without an emphasis on home production for own consumption. Six projects included activities focused on increasing market access to nutritious foods. At the same time, 36 projects reported an explicit focus on gender either through targeting of agriculture activities to women or SBC that engaged communities in women’s social and economic empowerment; none of the included projects reported activities directed at reducing women’s time burden or energy expenditures. Eleven projects reported activities related to food processing, storage, or preservation to enhance access and only one focused on time/labor saving through the provision of energy-efficient stoves. Approximately 85% of projects (*n* = 55) provided inputs beyond agricultural extension, education, and training. Inputs included seeds, saplings, vines, chickens, livestock, animal feed, fertilizers, and equipment.

### Effectiveness ratios for agriculture-to-nutrition pathways

Table 2 presents ERs for the different agriculture-to-nutrition pathways. Although all projects aimed to improve diet quality or diet diversity, 28 projects met our assessment criteria for estimating ERs. Of these, 18 assessed MDD-C, 17 assessed DDS-C, and 12 assessed WDD. Twenty-six of these 28 projects applied pathway 1 “increased production for home consumption” and of these, 21 applied additional pathways. Neither pathway #2 (increased production for sale) nor #5 (increased engagement of women in agriculture) were used on their own, rather they were layered onto other pathways, most commonly pathway #1. As expected, ERs decreased when restricting analyses to higher quality studies (RoB > 0.7). In an analysis restricted to studies with RoB > 0.7, increased production for home consumption demonstrated the highest ERs for MDD-C, DDS-C, and WDD (ER = 0.60, 0.64, and 0.43, respectively), compared with other pathways. This means that 60% of the projects that used pathway 1 with or without other pathways *and* reported MDD-C, and 64% of the projects that used pathway 1 with or without other pathways *and* reported DDS-C showed statistically significant improvements in those outcomes. Pathway #4, “increased access through reduced wastage,” had the highest overall ER and the highest ER for improving DDS-C. However, because this pathway was only used in 6 of the 28 projects, we were not able to calculate ERs for the other dietary diversity outcomes.

**TABLE 2:**
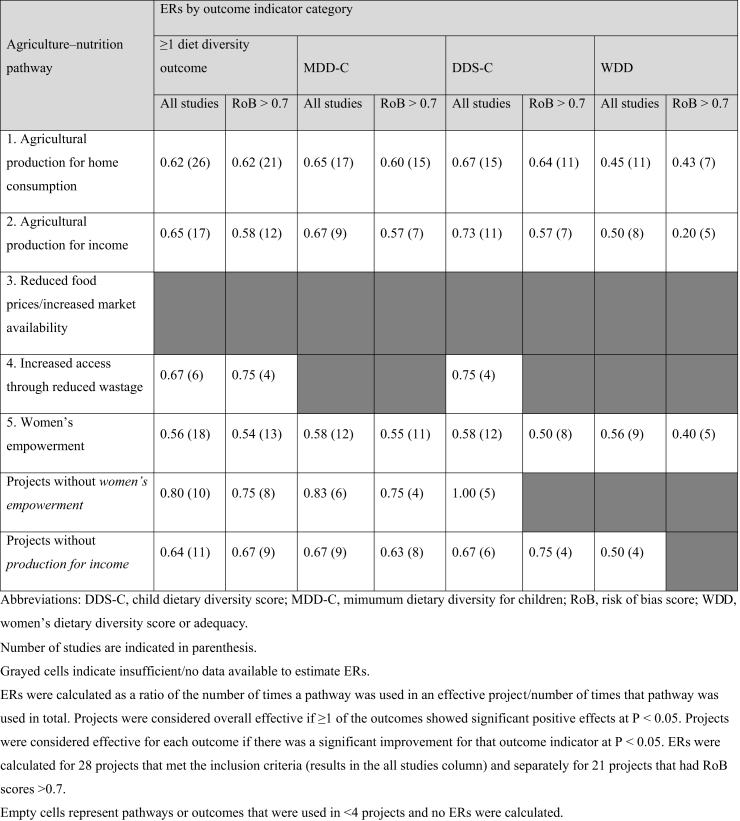
Effectiveness ratios (ERs) for agriculture-to-nutrition pathways overall and by diet diversity outcomes.

Projects were less effective at shifting women’s diet diversity with ERs ranging from 0.20 for pathway #2 to 0.43 for pathway #1, indicating that less than half of projects that targeted women’s diet diversity were effective at shifting this behavior. Interestingly, projects without a *women’s empowerment* component had higher ERs for all outcomes where calculations were possible compared with those that used women’s empowerment as a pathway to improve nutrition (Table 2).

### SBC approaches

**FIGURE 4:**
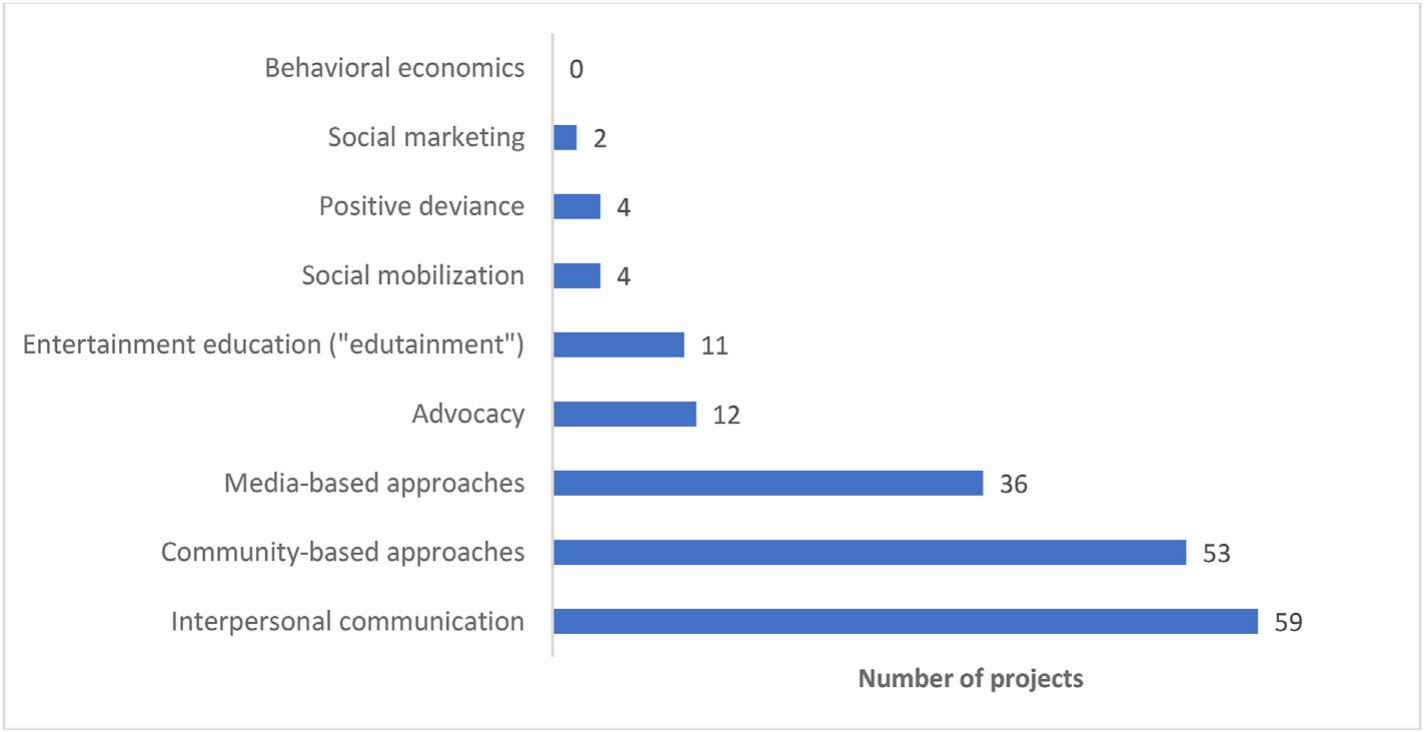
Social and behavior change approaches used by projects included in this review (*n* = 65). Definitions of each approach are included in OSM Supplemental Appendix C, Table C1.

IPC was reported in 59 of 65 projects while 53 projects reported using community-based approaches (Figure 4). Media was also frequently used, with 36 projects reporting some media usage (12 mass media, 22 small media, 13 mid-media, and 6 digital media). Fewer projects reported approaches such as advocacy (*n* = 12) or social mobilization (*n* = 4). Projects used, on average, 3 approaches, with 10 projects using only 1 approach and the maximum number used by any 1 project being 7.

### Effectiveness ratios for SBC approaches

Twenty-six of the 28 projects included in ER estimation used IPC. ER calculations conducted for the SBC approaches showed that IPC was not only the most frequently used approach but also the most effective overall (Table 3; ER = 0.65, ER RoB > 0.7 = 0.62), as well as for MDD-C (ER = 0.67; ER RoB > 0.7 = 0.60) (Table 3). Those that also included media-based approaches had the highest ERs for DDS-C (ER = 0.78; ER RoB > 0.7 = 0.67). The overall ER for projects that used >3 approaches (ER RoB > 0.7 = 0.69) in their SBC strategy was lower than those that used 3 or fewer approaches (ER RoB > 0.7 = 0.50). Using 3 or fewer approaches also demonstrated higher ERs for MDD-C and WDD (Table 3). The exception was DDS-C which had higher ER for projects using >3 SBC approaches.

**TABLE 3:**
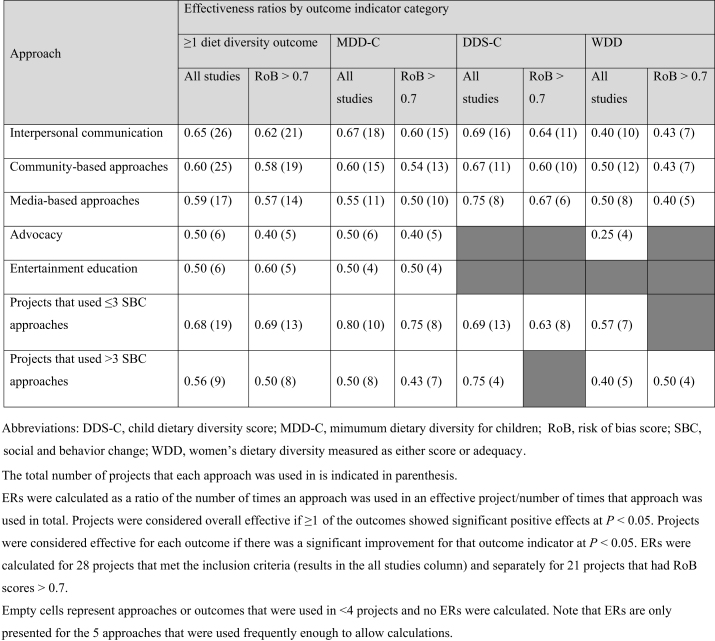
Effectiveness ratios for social and behavior change approaches effective overall and for each dietary diversity outcome of interest.

### Behavior Change Techniques (BCTs)

**FIGURE 5:**
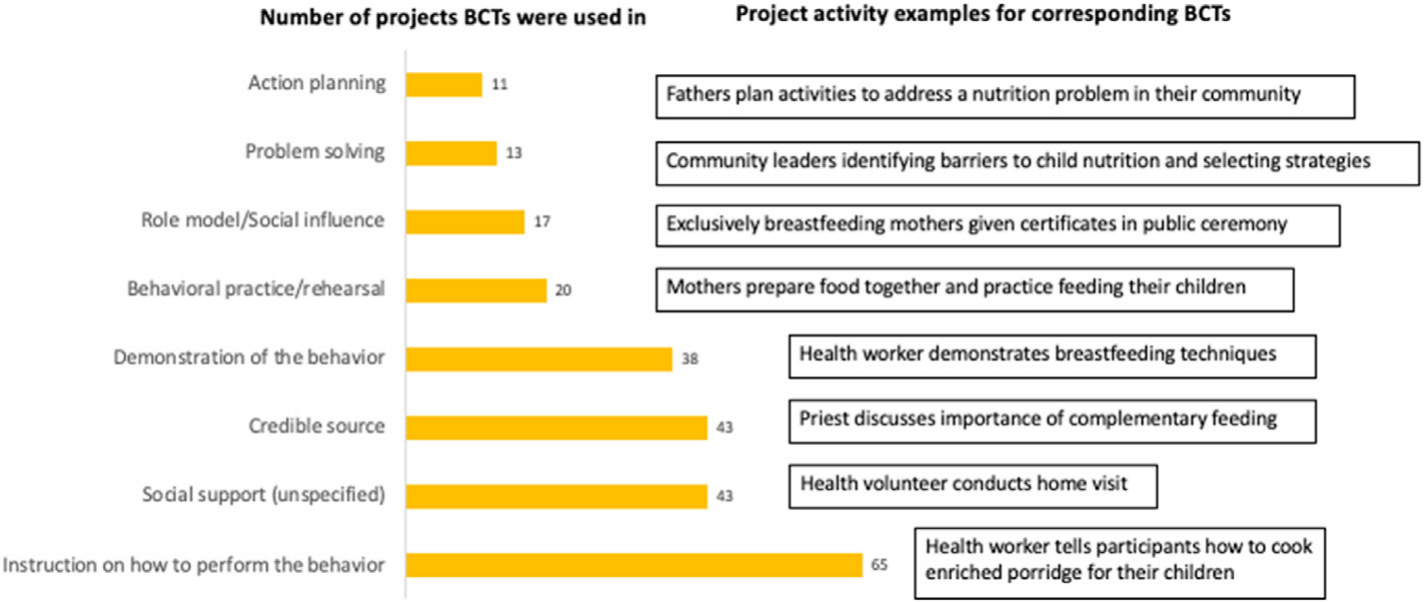
Counts and examples of commonly applied behavior change techniques (BCTs) used in interventions included in the systematic review (*n* = 65). Additional examples are provided in OSM Supplemental Appendix C, Table C2.

We identified 47 BCTs used in ≥1 project, 44 from the Taxonomy of BCTs [11], and 3 new BCTs developed by the research team (maintenance activities, role model/social influence, and promotion). Eighteen of these BCTs were used in 10 or more projects, while 11 were identified in only 1 intervention each. Figure 5 shows examples of more commonly used BCTs and the number of projects that used them (a longer list with definitions is available in online Supplemental Appendix C, Table C2).

Projects used on average, 8 unique BCTs in their SBC strategy, with the maximum number of unique BCTs used by any 1 intervention being 21 and the minimum being 1. BCT (4.1) “i*nstruction on how to perform the behavior*” was identified in all 65 included projects and was often coded when group or individual nutrition education activities were described. The other most frequently used BCTs were BCT (3.1) *“social* *support* *(unspecified)”* (*n* = 43), commonly used for individual counseling and household visits by frontline health or agriculture extension workers, and use of a “*credible source*” [BCT (9.1)] such as a trusted health worker, agriculture expert, religious leaders, or local celebrity for information dissemination (*n* = 43).

### Effectiveness ratios for BCTs

The most commonly applied BCT, (4.1) “*Instruction on how to perform the behavior*” had an overall ER of 0.64 (ER RoB > 0.7 = 0.62) with higher ERs observed for MDD-C and DDS-C indicators compared with women’s diet diversity (Table 4). For child diet diversity indicators (MDD-C or DDS-C), 4 BCTs had ERs >0.75 when ER analyses were restricted to higher quality studies (RoB > 0.7). These included 1.4 “*Action planning,*” 6.1 *“Demonstration of the behavior,*” 8.1 “*behavioral practice,*” and 9.1 “*credible source*.” Indeed, with an ER of 1.0, all projects that used the BCT 8.1 “*behavioral practice*” significantly improved child diet diversity. No BCTs achieved ER > 0.6 for women’s diet diversity indicators. In looking at combinations of BCTs, projects using ≥7 BCTs had higher ERs than those that used <7 BCTs for all outcomes except WDD. We did not have a sufficient number of studies to examine the ERs of specific combinations of BCTs for diet diversity outcomes.

**TABLE 4:**
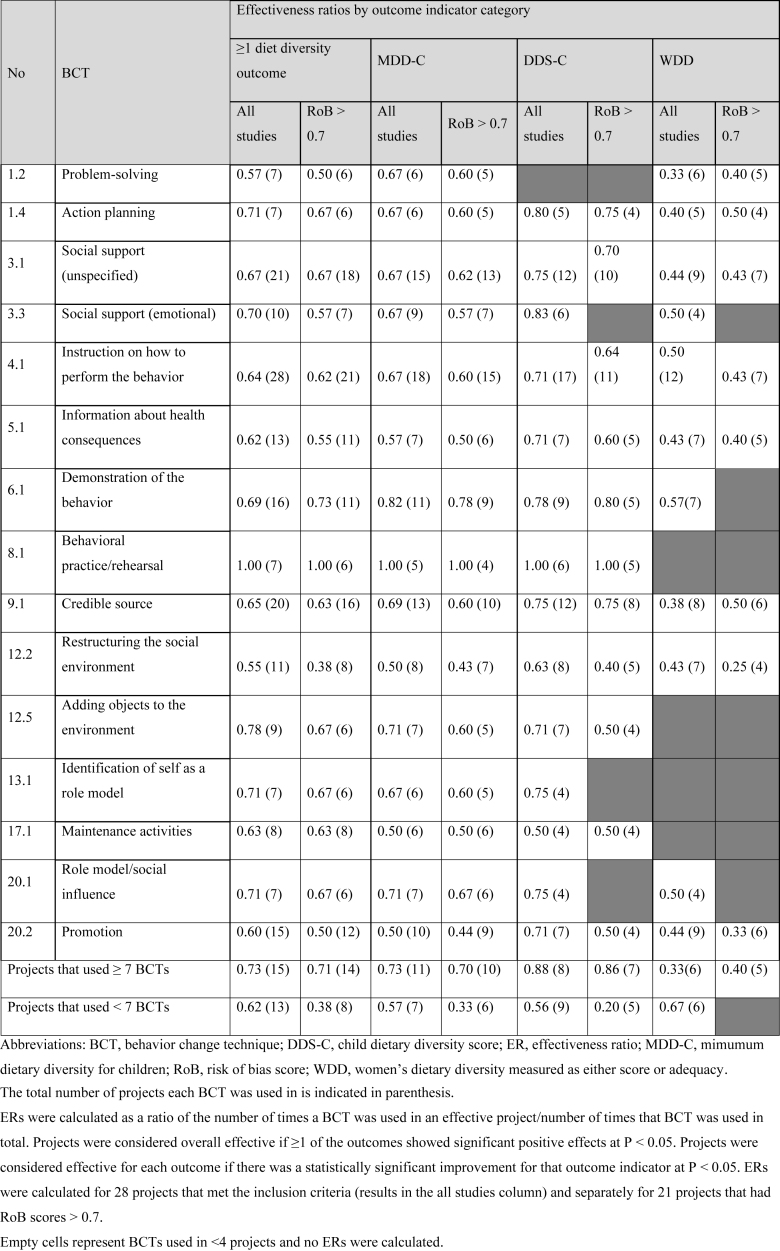
Effectiveness ratios for Behavior Change Techniques (BCTs) that were effective overall and for each dietary diversity outcome of interest.

## Discussion

Extensive and thoughtful work has been done to enhance and promote effective nutrition behavior change at scale (see, for example, resources prepared by SPRING, FSN Network, USAID, and ANH Academy) including, more recently, in the context of agriculture. However, reviews of nutrition behavior change projects focused on anemia prevention, maternal nutrition, and IYCF consistently document that the content, design, and implementation of these interventions to improve diets and nutrition is highly variable [16,[32], [33], [34], [35]]. Consistent with our own findings, these reviews also highlight how few projects apply commonly agreed-upon best practices for SBC, including, for example, *1*) the use of formative research to inform program design and implementation, *2*) the application of evidence or theory-informed intervention design frameworks or behavior change theory, and *3*) utilization of theory of change or program impact pathways to delineate how programs will achieve change. And, as noted by others [36], the systematic documentation and sharing of behavior change strategies as they relate to content and delivery is rare, thus limiting our understanding of not only *if*, but also *how* interventions affect dietary behavior change. These limitations in the evidence base challenge researchers and programmers seeking to identify, replicate, and adapt evidence-based strategies and approaches for further testing or scale-up.

This review on nutrition behavior change strategies used in NSA projects, with its emphasis on the interventions themselves, aims to strengthen our understanding of “*how*” programs may effect change. Our review identified similar gaps as those identified in previous reviews [16,[32], [33], [34], [35], [36]] and 3 key takeaways unique to NSA: *1*) the majority of NSA interventions included in this review focused on the agriculture-to-nutrition pathway of increasing the availability of nutritious foods for consumption through the production of these foods at home; other, less frequently used pathways, such as reducing wastage show promise and need further investigation for effectiveness; *2*) IPC was both the most frequently used SBC approach and the most effective at improving dietary diversity indicators; and *3*) More participatory BCTs, for example, those that engage participants in action planning, demonstrations, behavioral practice, and use credible sources to facilitate SBC activities were more commonly found in interventions that significantly improved child diet diversity indicators.

The majority of projects layered other agriculture-to-nutrition pathways onto pathway #1 “agricultural production for own consumption” so it is challenging to disentangle whether improvements were related to increased production, increased income, women’s empowerment, or some combination of these. Hypothetically, programs that increase total household income in resource-poor settings increase consumption of both food (Engel’s law) and other goods and services that contribute to health and nutritional status [37]. A dual pathway approach that both increases own production for consumption and generates income allows for greater economic flexibility and resilience. Income can be invested into agricultural inputs and savings, used to support other household health and well-being needs, or to purchase foods that are not or cannot be produced by the household. Indeed, social safety net strategies that increase agricultural income may be most effective at reaching poorer households that depend on agriculture livelihoods and hence best suited for poverty reduction and nutrition improvement in these contexts [38]. Further data compiled from the World Bank, Living Standards Measurement Survey demonstrate that, even among rural consumers in low-income, households procure 40%–80% of their food from markets as opposed to home production [39]. Thus, nutrition-sensitive strategies that mobilize agriculture for income generation either as a strategy on its own or in combination with other pathways may have greater impacts on dietary diversity; particularly if sufficient attention is given to creating demand for nutritious foods.

Women’s empowerment has long been a focus of maternal and child nutrition programs. Although the evidence for associations between women’s empowerment and child stunting or wasting are mixed [40], interventions that seek to engage women in and benefit from agriculture appear to improve women’s and children’s diets via multiple pathways including improved spousal and social communication skills, engagement in decision making, and greater autonomy [[41], [42], [43], [44]]. Furthermore, evidence suggests that empowering women with equal access to productive inputs, including land, could increase farm yields by 20%–30%, raising total agriculture output by 2%–4% and reducing undernourishment by 17%–19% [45]. Of those included in ER calculations, 18 projects utilized pathways that address women’s empowerment in agriculture. Most commonly, interventions engaged women in agricultural activities, providing inputs and access to land to increase women’s control over income. Interestingly, interventions included in ER estimations that acted on the women’s empowerment pathway had the lowest ERs for child dietary diversity outcomes. Research notes that women’s empowerment activities can carry unintended consequences of increased burden on women’s time and labor and potentially undermine possible benefits if these unintended consequences are not taken into consideration [6,[46], [47], [48]]. None of the interventions included in this review reported monitoring for or implementing activities to mitigate this unintended consequence and no projects had an explicit focus on reducing women’s time/energy burden.

Reducing food loss had the highest ER of the pathways examined when considering all of the diet diversity outcomes and for DDS-C; however, this analysis is limited by the small number of projects applying this approach. Although 11 projects addressed the food loss pathway, often in conjunction with increased production for own consumption, only 6 reported outcome data and contributed to ER analysis. These projects focused efforts predominantly on food processing and storage practices. The Food and Agricultural Organization estimates that ∼14% of food is lost after harvesting but before reaching markets as a result of inadequate processing, storage, handling, or transportation. Estimates vary depending on commodity type, but more nutritious foods such as animal-source foods, fruits, and vegetables are more susceptible to postharvest loss [49]. Food loss is recognized as a significant impediment to economic and food security in contexts dependent on agricultural livelihoods. Although attention in this area is growing, the evidence base for effective on-farm practices to reduce loss, especially among poor households, and how those activities translate into improved food security and diet quality, remains limited.

A large majority of the projects used SBC approaches that depend on IPC, for example, through one-on-one interactions with frontline workers or through small group activities. Community-engaged approaches as well as media were also commonly used. On the contrary, advocacy-based approaches were rarely reported. Previous research highlights the effectiveness of both mass media and advocacy to improve women’s diets, complementary feeding, and breastfeeding behavior change [32,50]. Approximately half of the projects in this review that were included in ER estimations utilized >2 approaches. The layering of multiple approaches is considered an evidence-based best practice for SBC [51]; however, this best practice implies that teams are capacitated to implement multiple approaches well. In our analysis, those interventions using >3 approaches had lower ERs for most diet diversity outcomes compared with those using 3 or fewer approaches. Although we have limited data on the quality of implementation, it is plausible that if projects were not sufficiently capacitated to implement multiple approaches, they may have experienced a dilution effect, needing to spread scarce resources and capacity across multiple activities, ultimately reducing the quality of implementation overall. Indeed, this could also be one explanation for the difference in ER for diet diversity outcomes between projects that included activities focused on the women’s empowerment pathway from agriculture-to-nutrition compared with those that did not. If projects experience competing priorities or are not adequately capacitated and funded to implement these approaches well then effectiveness may suffer, particularly if the emphasis or quality of SBC focused on diets and nutrition is suboptimal.

Limited research has examined the techniques used in diet-related behavior change programming in LMICs [16]. The BCTs most commonly applied in studies reviewed here were those that are educational or information dissemination in nature – for example, providing instructions, promotion, information on consequences, and emphasis on the information source (that is, “credible source”). Of this type of BCTs, none had ERs > 0.80. More participatory BCTs, for example, related to behavioral demonstration, behavioral practice, and action planning, were less frequently applied but had higher ERs for both children’s and women’s diets. These findings are similar to what we observed in our work on BCTs in complementary feeding [16].

Reviews of nutrition interventions in high-income settings that focus on diet change for chronic disease [12,13,19,52] note that effective studies are more likely to explicitly define a behavior change theory and select techniques that address behavioral determinants (identified through formative research) within these theoretical constructs. Examples of BCTs seen in these effective studies included “environmental restructuring,” “goal setting,” “social support,” and “behavioral practice/rehearsal.” Unfortunately, a few of these more participatory and effective approaches were rarely used in studies included in this review and could not be included in ER calculations.

Systematic approaches to aid SBC designers in using theory, identifying key behavioral determinants, and bridging BCTs to behavioral determinants include Intervention Mapping, Designing for Behavior Change, and The Behavior Change Wheel approaches. These approaches often depend on robust formative research. Consistent with a previous review on interventions to improve complementary feeding of infants in LMICs [16], while most projects in this review conducted formative research, very few specified a guiding behavior change theory. Only 13 studies included in this review specified the application of a systematic approach to their SBC design, including 2 that applied the Designing for Behavior Change approach. These findings suggest a general lack of a systematic and strategic approach to SBC design for nutrition which may contribute to reduced effectiveness. Resources and tools exist to guide SBC implementers in conducting formative research and applying theory-informed, systematic, and strategic design tools, including for NSA (see, for example, Tools and Resources from USAID Advancing Nutrition, USAID SPRING, and the Agriculture, and Nutrition and Health Academy).

In this review, the BCT “social support” (unspecified), was the second most frequently used BCT and had an overall ER of 0.67 for studies with RoB > 0.7. This code was applied, per the definition, to activities that included counseling (one-on-one or in small groups) and peer encouragement and is consistent with the finding that IPC was the most frequently used SBC approach. Social support strategies, particularly those that are family-centric and encourage the engagement of husbands and grandmothers to support diet change are increasingly recognized as effective to improve maternal and child nutrition [[53], [54], [55], [56], [57], [58]]. Other social support strategies included projects ranging from “practical” support (for example, women forming savings groups to achieve financial goals) to “emotional” support where target groups received advice on nutrition behaviors (for example, intergenerational conversations about child feeding experiences). Increasingly, maternal and child nutrition research supports family-centric SBC approaches to change behaviors and attitudes of key influencers, for example, husbands and grandmothers [59]. It is believed that doing so creates an enabling family or community environment for change with regard to social and gender norms that influence maternal and child nutrition [60,61]. Integrating BCTs into family-centric models that allow behavior change strategies to effectively target multiple levels of the family and social-ecological model and that build on adult education theory per the needs, culturally designated roles, and responsibilities of those target groups may be an effective strategy to support diet-related behavior change. For example, using BCTs such as problem-solving, goal setting, and action planning with fathers or self as role models, demonstration, behavioral practice, and social support with grandmothers may shift perceptions and behaviors of these key influencers in the household creating a more enabling family environment for behavior change for mothers and children. However, examples of this work are limited in the nutrition space and are an area in need of further research. In this review, less than half of the projects (*n* = 30) explicitly reported SBC strategies to engage husbands, grandmothers, or other family members in their project materials, 14 of which qualified for inclusion in ER estimations. Of these, 13 measured child diet diversity with 10 reporting significantly positive effects [[62], [63], [64], [65], [66], [67], [68], [69], [70], [71], [72]]. Seven measured women’s diet diversity of which 3 reported significantly positive effects [64,72,73]. This points toward an increasing need to explore social support strategies in NSA projects, especially in improving child nutrition outcomes.

Both the proportion of projects measuring women’s dietary outcomes and the proportion achieving significant improvements in women’s diet diversity outcomes were lower than that for child dietary diversity. This may suggest a lower priority for women’s diet and nutrition outcomes in NSA interventions relative to children’s. Lower prioritization may result in less capacitation and funding or fewer activities for SBCs focused on women’s diets. Additionally, less research has historically been conducted on SBC and educational interventions to improve women’s diets compared with those of young children. This body of work predominantly focuses on pregnant and lactating women, a narrow window of a woman’s lifecycle. Thus, fewer resources, experiences, and materials exist to guide the development of SBC interventions aimed at improving women’s diets. As well, and as seen in our review of the material provided for this work, maternal and child nutrition is often combined into a single “package” of SBC activities utilizing the same approaches and techniques. It is not implausible that the SBC approaches and techniques necessary to spur women to change their own diets (and communities to support these changes) differ drastically from the approaches and techniques needed to effect change in children’s diets.

### Strengths, limitations, and implications

A major limitation in this type of analysis is that we must rely on and code information as it is reported in documents. We are unable to verify, based on this reported information, what SBC approaches and techniques were ultimately used, the fidelity of the program activities to the stated protocols, or the quality of implementation. Per the protocol outlined by the BCT taxonomy training, only those BCTs with sufficient detail on a given technique could be coded for that BCT. Too often, peer-reviewed manuscripts and publicly available reports provided insufficient detail to code specific BCTs. Despite contacting corresponding authors, we were unable to locate additional details for ∼35 of the included projects. As such, we likely underreport BCTs as we were constrained by the coding approach to code as absent those techniques that intervention designers may argue were present but were not reported. It is also plausible that activities reported in project documents and reports may not have been implemented (for example, loss of fidelity) which may create an overestimation of BCT utilization and effectiveness. The ability to reproduce interventions depends on understanding the details of the intervention and its implementation. The inability to code due to insufficient detail also reduces the ability to develop more robust ERs and increases the risk of over or underestimating ERs for those BCTs with fewer studies. To mitigate this, we limited our analysis to BCTs used in 4 or more projects. It should be noted that the number of interventions used as a threshold for inclusion varies across studies from 3 [12] to 5 [16,19]. However, given the limited number of studies overall, when applying a higher threshold, we lose the ability to analyze BCTs that have historically been associated with effectiveness in other diet-related interventions [12,16]. These include techniques related to action planning and practice/rehearsal of the behavior, both of which had high ERs in our analysis.

Significant heterogeneity existed across studies with respect to agricultural activities, behavior change approaches, BCTs, and outcome measures. Although such heterogeneity may imply that programs are designing contextually appropriate interventions, such variation in the active intervention components makes comparison of studies challenging and meta-analyses of impacts potentially inappropriate [74]. As such, we applied the ER approach to characterizing the active components that were commonly seen in effective interventions as opposed to calculating meta-analytic estimates of impact for NSA interventions. This approach may have greater utility when thinking more granularly and qualitatively about the design and implementation of NSA interventions, which are often complex and multifaceted. Although we acknowledge that there are limitations to ERs, we argue they provide a more qualitative assessment of the potential effectiveness of the individual components of a larger intervention and may be more useful in implementation science research to understand the “how” of an intervention's effectiveness, help identify promising intervention components and identify components that may be noncontributory. To overcome limitations to the use of ERs we limited estimations to studies with more robust evaluation designs and that measured children’s or women’s diet diversity indicators to minimize heterogeneity. This approach reduced our sample for ER estimation from 65 to 28 studies. Although we acknowledge that this reduction likely introduces bias, and may cause us to miss potentially promising approaches or BCTs or over/under estimate ERs, we took this more conservative approach to improve confidence in the ERs produced.

Projects used a range of different diet-related indicators as outcomes, including minimum diet diversity, diet diversity scores, micronutrient and macronutrient intakes, nutrient adequacy ratios, consumption of specific food groups, and consumption of specific foods. In addition, a substantive minority of projects (*n* = 28) utilized household-level indicators as a measure of individual dietary diversity, including, for example, household diet diversity scores, household production diversity, and household food consumption patterns. Seven only reported household-level indicators. Given gender and age-discriminatory household food allocation patterns in many contexts, household-level indicators are considered more appropriate proxies for household food security than women’s or children’s diet diversity. Additionally, indicators utilized different approaches and recall periods including, for example, 24-h quantitative recalls, 7–30-d food frequency questionnaires, food consumption surveys, and expenditure surveys. To reduce heterogeneity and maximize comparability across projects we used individual diet diversity indicators (for example, minimum diet diversity and diet diversity scores) as the outcome measures for ERs. Diet diversity indicators were the most commonly used indicators and more consistently applied a standardized or validated approach such as that described by the FAO or WHO. The large majority of these utilized a 24-h recall period. We recognize the exclusion of outcomes such as micronutrient intakes or food groups may introduce bias into the ER measure and reduce the sample size of projects for ER calculations.

Another critical limitation in our review was the inability to assess specific implementation details including, for example, programming costs, participation in project activities (for example, dose), and SBC implementation fidelity. These details were rarely reported in peer-reviewed manuscripts, publicly available gray-literature documents, or documents provided by implementers on request. Although it was beyond the scope of this work to track these data and incorporate them into this review, prior work has noted that quality SBC implementation is a critical bottleneck in NSA [6]. This work and others highlight the potential for low-quality SBC implementation, particularly in low-resource areas where interventions may not be contextually appropriate for the stated aims in the given context, participants may not have the capacity to initiate or sustain behaviors or community-based frontline workers (typically volunteers) are tasked with implementing SBC activities without adequate compensation, training or supportive supervision [[75], [76], [77], [78]]. Furthermore, research has noted that NSA is perceived to have higher costs than nutrition-specific interventions [75]. However, the limited research on the cost and cost-effectiveness of NSA in general [6,75] and the specific contributions of different agriculture-to-nutrition pathways, SBC approaches and activities remains a significant gap in this field that limits implementers’ ability to select cost effective approaches for a given context.

NSA, nutrition, and diet-related behavior change work more broadly, are areas of intense focus and investment for global development. Of particular interest is programming at scale. However, replication of effective nutrition behavior change interventions to both verify impacts and enable scale-up has proven challenging. Programs rarely report intervention design frameworks, theories of change, or implementation details to allow bridging of activities to specific changes in behavior and assessment of effectiveness. This lack of reporting (and potential application) is a recognized gap for both nutrition-specific and nutrition-sensitive interventions [6,16,32,33,36]. Comprehensive guidelines exist for reporting behavior change interventions [79,80] including a checklist for reporting on group behavior change interventions, such as those commonly seen in NSA. However, currently, only those interventions that self-identify as clinical or randomized controlled trials are held to a higher reporting standard and required to input such details into an open-access registry (for example, clinicaltrials.gov); others do so voluntarily. To address these gaps, more detailed, systematic open access, and transparent documentation of NSA projects, including their SBC components, will be critical not only for identifying what works, does not work, and why, but also to enable replication, adaptation, and scale-up of strategies and approaches that do work. Design and implementation details and SBC strategies should be required as online supplemental materials for journal publication. Alternative affordable, open-access platforms that enable standardized dissemination of SBC strategies and implementation processes should also be made available to projects that are unable to engage in the peer-reviewed publication or trial registry process. These reporting mechanisms can support donors, implementing agencies and research institutions to review and evaluate relevant implementation details.

In conclusion, nutrition SBC programs delivered through agricultural platforms have been developed by a multitude of non-governmental organizations (NGOs), government ministries, universities and research institutes, and food and agricultural companies. These programs vary widely in terms of the agriculture strategy within which they are embedded, the SBC approaches used, and the specific BCTs applied. Based on our results, we outline the following recommendations:

• Less frequently utilized agriculture-to-nutrition pathways may hold promise in improving the diets of women and children. Further testing of the effectiveness of these pathways, such as reducing waste, is needed.
• Women’s empowerment is recognized as a primary pathway to improved nutrition; however, careful consideration is needed when designing activities that act along this pathway. Programs should strive for high-quality implementation of gender transformative approaches and ensure women’s engagement in program activities does not adversely affect their time and labor burdens, energy expenditure, or other health risks.
• Although layering SBC approaches is a recommended practice, using too many approaches may stretch project resources and dilute project effects. Programs should consider a manageable number of approaches based on budget and capacity. Conducting robust formative research, selection, and application of behavioral theory and utilization of evidence-based intervention design frameworks may aid programs in ensuring effective and feasible layering of approaches.
• BCTs that are more participatory in nature are underutilized but show potential for effectiveness. Additional research is needed to test these techniques with the goal of greater integration into SBC intervention design and implementation. Simultaneously, greater attention to and documentation of the costs of different techniques and approaches to inform selection is needed.
• Increased prioritization of women’s diet before, during, and after pregnancy and lactation is needed, along with explicit research to identify barriers to and drivers of diet choices among women. Such actions will support the identification and testing of promising approaches and techniques to improve women’s diets.

## Author contributions

The authors’ responsibilities were as follows – AWG: designed the research and supported article review for inclusion, resolving coding discrepancies and supported data analysis; TT, LSB, CXE: conducted the systematic search, abstracted and coded data; TT, AWG: analyzed the data; AWG, TT: wrote the article; AWG: had primary responsibility for final content; and all authors: read and approved the final manuscript.

## Conflict of interest

The authors report no conflicts of interest.

## Funding

This work was funded by the Bill and Melinda Gates Foundation INV-001984. The funder had no involvement in or restrictions on the published work.

## Data availability

Data described in this manuscript will be made available upon request pending approval.
