# Supplementary material for: Effectiveness of Elements of Social Behavior Change Activities in Nutrition-Sensitive Agriculture Programs: A Systematic Review
Source: Curr Dev Nutr. 2024 Jul 26;8(8):104420. doi: 10.1016/j.cdnut.2024.104420 (PMC11367542; doi:10.1016/j.cdnut.2024.104420)

**APPENDIX A**

**Agriculture to Nutrition Pathways and Descriptions (adapted from Herforth & Harris. 2014 and Duncan et al. 2022)**

| **Agriculture-Nutrition Pathway** | **Description** | **Examples of intervention activities** |
| --- | --- | --- |
| Agricultural production | Production of nutritious foods by those with access to arable land with the objective of consuming some of that produce in the home. Increased agricultural production of nutritious foods increases food security by making foods available year-round and reducing reliance on market prices and availability | Encouraging participants to grow kitchen or community gardens of nutritious foods such as green leafy vegetables or orange fleshed sweet potatoes for (at least partial) household consumption. |
| Agricultural income | Generating or increasing income from agricultural activities (i.e. agribusinesses, small farms etc) allows increased spending on nutritious foods available in the market, ultimately leading to increased consumption in households. | Poultry cooperatives that organize women’s groups to rear chickens, sell eggs for profit, and use the income to purchase other nutritious foods for their children. |
| Women’s empowerment | Increasing women’s decision-making power, control of assets (including finances), and agency/autonomy leads to improved. May also include aspects related to women’s time use, energy expenditures and nutritional status | Women’s community gardens that produce crops for sale, giving women additional income and control on spending of that income, time / labor saving devices. |
| Market availability | Increasing the availability and accessibility of nutritious foods in the market ensures that communities can purchase foods, improving household nutrition. | Providing seeds of biofortified crops to large producers to grow, propagate, and sell in the local market. |
| Food processing and reduced wastage | Processes that increase efficiency of production, and utilize proper transportation and storage reduce food waste. These processes can decrease contamination and increase shelf life, increasing the availability of nutritious foods in households or communities. | Community granaries that store grains that vulnerable community members can access during dry or low seasons. |

**Agriculture to Nutrition Pathways Model (Herforth and Harris, 2014)**


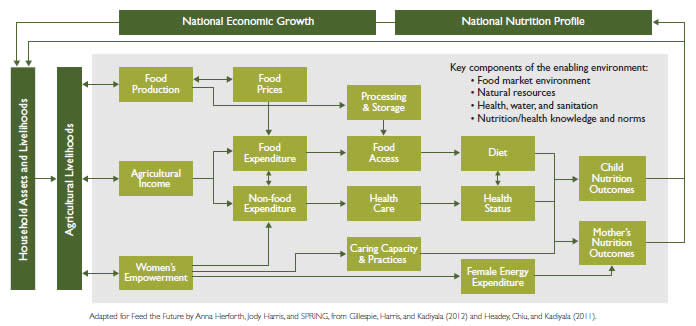

Supplement: Multimedia component 1 [file mmc1.docx]
