# Supplementary material for: Effectiveness of Elements of Social Behavior Change Activities in Nutrition-Sensitive Agriculture Programs: A Systematic Review
Source: Curr Dev Nutr. 2024 Jul 26;8(8):104420. doi: 10.1016/j.cdnut.2024.104420 (PMC11367542; doi:10.1016/j.cdnut.2024.104420)
Supplement: Multimedia component 3 [file mmc3.docx]

**OSM - APPENDIX C – SBC Approaches and BCTs**

| ***Table C1***. Social and behavior change approaches used in the 65 unique projects included in this review. Projects may have used more than one approach. | | |
| --- | --- | --- |
| **Approach** | **Definitions of approaches** | **Number of projects** |
| Interpersonal communication | Two-way, direct, personal communication, generally face-to-face.* Includes one-on-one or interactions between groups small enough for conversation. | 59 |
| Community-based approaches | Approaches that engage the broad community in various ways to achieve social change, inspire collective action, or disseminate information relevant to the behaviors of interest. Projects that work with the community as a whole, engage with specific organized groups, and/or involve local leaders would be included in this category. This is an umbrella term that includes community engagement, mobilization, outreach, and dialogue. | 53 |
| Media-based approaches | This includes mass media (broadcast media that reaches a wide audience), mid-media (distributed through a small, defined geographic area such as community radio, public address, billboards), small media (distributed to individuals and small groups such as counseling cards, flyers, stickers), and digital/social media (using the internet or cell phone networks). | 36 |
| Advocacy | Communications and activities designed to raise awareness, shape policy, stir commitment to program objectives and mobilize resources | 12 |
| Entertainment education ("edutainment") | Entertainment is performed live in community spaces or broadcast on TV or radio. Performance is designed to educate and entertain simultaneously. Generally one-way communication, but can be combined with discussion (IPC) for deeper impact. | 11 |
| Social mobilization | More focused on affecting the enabling environment, broader socio-structural change. Brings stakeholders, leaders, and communities together to raise awareness and mobilize action. | 4 |
| Positive deviance | A specific method for promoting behavior change in communities that cultivates as community assets those individuals who practice uncommon but healthy behaviors and who help peers try successful practices through practical learning and problem solving | 4 |
| Social marketing | The application of marketing methods and concepts to promote products, services or behaviors for health or a social good. Highlights the audience perspective and uses a mix of media to address needs and wants to achieve objectives. | 2 |
| Behavioral economics | Approaches drawing on psychology and economics use changes in the physical environment and ‘nudges’ to trigger behavioral choices sometimes subconsciously. | 0 |
| *Telephone or text conversation is two-way, direct communication but not face-to-face, but is considered IPC. Note: Some IPC may not *actually* include much two-way dialogue, but if its stated *intention* is to be interactive and the number of people allows interaction (generally < 15 people), it is considered IPC. | | |

| ***Table C2*:** The most frequently identified Behavior Change Techniques in the 65 nutrition-sensitive agriculture projects included in this review. This table outlines BCTs used in 10 or more projects, their definitions from the BCT Taxonomy^1^, the number of projects that used each BCT, and a short example from an included study that was coded with that BCT. | | | | |
| --- | --- | --- | --- | --- |
| **BCT Number** | **BCTs** | **Definitions from taxonomy** | **N** | **Examples from projects included in this study** |
| 4.1 | Instruction on how to perform the behavior | Advise or agree on how to perform the behavior (includes ‘Skills training’) | 65 | *Pregnant/breastfeeding mothers are given training on breastfeeding techniques and complementary foods to give at different stages of child development* |
| 3.1 | Social support (unspecified) | Advise on, arrange or provide social support (e.g., from friends, relatives, colleagues,’ buddies’ or staff) or noncontingent praise or reward for performance of the behavior. It includes encouragement and counselling, but only when it is directed at the behavior Note: attending a group class and/or mention of ‘follow-up’ does not necessarily apply for this BCT, support must be explicitly mentioned | 43 | *Situation appropriate nutrition counseling provided to mothers during household visits or in small groups at health centers*  Note that this code was most often used for one-on-one counseling activities. |
| 9.1 | Credible source | Present verbal or visual communication from a credible source in favour of or against the behavior Note: code this BCT if source generally agreed on as credible e.g., health professionals, celebrities or words used to indicate expertise or leader in field and if the communication has the aim of persuading | 43 | *Farmers were trained on infant and young child feeding and diet diversification by male agriculture and nutrition experts in the community.* |
| 6.1 | Demonstration of the behavior | Provide an observable sample of the performance of the behaviour, directly in person or indirectly e.g., via film, pictures, for the person to aspire to or imitate (includes ‘Modelling’) | 38 | *Participants were taught how to use available ingredients to prepare different recipes for the family meal or complementary feeding of infants.* |
| 20.2 | Promotion* | Use this code for social marketing, promotion activities and sensitization campaigns. These often target a broad social group (for example entire communities or all farmers). This is meant to serve as a blanket code for such activities | 33 | *Activities promote specific crops for home consumption and are delivered by agriculture extension workers.* |
| 5.1 | Information about health consequences | Provide information (e.g., written, verbal, visual) about health consequences (positive or negative) of performing the behavior Note: consequences can be for any target, not just the recipient(s) of the intervention; emphasizing importance of consequences is not sufficient | 28 | *Participants learned that exclusively breastfeeding a child without giving any water will reduce the possibility of infections and diarrhea.* |
| 12.2 | Restructuring the social environment | Change, or advise to change the social environment in order to facilitate performance of the wanted behavior or create barriers to the unwanted behavior | 26 | *Discussions among groups of fathers or grandmothers about the roles of women/mothers in society and male support with household chores*  Note: most activities coded with 12.2 were related to addressing and changing gender norms |
| 3.3 | Social support (emotional) | Advise on, arrange, or provide emotional social support (e.g., from friends, relatives, colleagues, ‘buddies’ or staff) for performance of the behavior | 21 | *Women’s groups were used to hold discussions about the challenges of breastfeeding and complementary feeding and provide a platform for women to share their own experiences.*  Note: for this study, code 3.3 was used for experience sharing and community discussion activities |
| 8.1 | Behavioral practice/ rehearsal | Prompt practice or rehearsal of the performance of the behavior one or more times in a context or at a time when the performance may not be necessary, in order to increase habit and skill | 20 | *Have mothers prepare complementary foods together, feed them to the child during the session, and watch their reaction.* |
| 12.5 | Adding objects to the environment^+^ | Add objects to the environment in order to facilitate performance of the behavior Note: Provision of information (e.g. written, verbal, visual) in a booklet or leaflet is insufficient | 18 | *Give each family a small bowl for feeding their child under two years that cues age-appropriate meal frequency and quantity*  *Providing posters or flyers that families could post in their homes and refer to after education sessions*  Note: for this study, posters/leaflets used as visual reminders of the behavior were included in this code. This code does not include agricultural inputs. |
| 17.1 | Maintenance activities^*^ | This code should be used for any activities conducted to maintain and/or reinforce behavior after the initial introduction of the behavior | 18 | *Follow-up visits were used as an opportunity to remind mothers of the messages on healthy nutrition and diet behaviors* |
| 13.1 | Identification of self as role model | Inform that one's own behavior may be an example to others | 17 | *Community members select lead women farmers to be responsible for maintaining demonstration gardens and training other women’s groups on agriculture and nutrition.* |
| 20.1 | Role model/Social influence^*^ | Use this code for activities where community members practicing a behavior influences others. This could include model community members, community champions or any activities where positive deviance was used. | 17 | *A graduation ceremony is conducted for mothers who have completed optimal IYCF behaviors when their child reaches two years old. All women in the community, especially pregnant and breastfeeding mothers are invited to the ceremony.* |
| 3.2 | Social support (practical) | Advise on, arrange, or provide practical help (e.g., from friends, relatives, colleagues, ‘buddies’ or staff) for performance of the behavior | 14 | *Establishment of savings and loans groups to promote nutrition practices and use savings to purchase small livestock for household consumption* |
| 1.2 | Problem solving | Analyze, or prompt the person to analyze, factors influencing the behavior and generate or select strategies that include overcoming barriers and/or increasing facilitators (includes ‘Relapse Prevention’ and ‘Coping Planning’) Note: barrier identification without solutions is not sufficient | 13 | *Monthly group discussions and community-based dialogue about social and gender inequalities within that community and discussed ways to address them*  Note: most interventions using participatory learning and action design were coded with 1.2 and 1.4 |
| 1.4 | Action planning | Prompt detailed planning of performance of the behavior (must include at least one of context, frequency, duration and intensity). Context may be environmental (physical or social) or internal (physical, emotional or cognitive) (includes ‘Implementation Intentions’) | 11 | *Community members plan feasible strategies to address the problems they identified, decide on roles and responsibilities for implementing the strategies, and discuss next steps*  Note: most interventions using participatory learning and action design were coded with 1.2 and 1.4 |
| 4.2 | Information about antecedents | Provide information about antecedents (e.g., social and environmental situations and events, emotions, cognitions) that reliably predict performance of the behavior | 11 | *Community leaders discuss root causes of malnutrition issues and suboptimal nutrition behaviors in their community*  Note: this code was mostly used for activities that teach/train participants about the causes of certain behaviors or health outcomes |
| ^1^ More detail on the BCT taxonomy can be found at Michie S, Richardson M, Johnston M, Abraham C, Francis J, Hardeman W, et al. The behavior change technique taxonomy (v1) of 93 hierarchically clustered techniques: building an international consensus for the reporting of behavior change interventions. Ann Behav Med. 2013;46(1):81-95. Epub 2013/03/21. doi: 10.1007/s12160-013-9486-6. PubMed PMID: 23512568 and  <https://www.bct-taxonomy.com/> | | | | |
