# Supplementary material for: Effectiveness of Elements of Social Behavior Change Activities in Nutrition-Sensitive Agriculture Programs: A Systematic Review
Source: Curr Dev Nutr. 2024 Jul 26;8(8):104420. doi: 10.1016/j.cdnut.2024.104420 (PMC11367542; doi:10.1016/j.cdnut.2024.104420)
Supplement: Multimedia component 6 [file mmc6.docx]

**OSM – APPENDIX E – Project Citations**

| **Table E: Characteristics of the 65 studies included and outcomes reported; *indicates studies included in effectiveness ratio estimation. Unless otherwise indicated child diet diversity indicators are reported for children ages 6-24m. Projects with an * met criteria for inclusion in effectiveness ratio estimation** | | | | | | | |
| --- | --- | --- | --- | --- | --- | --- | --- |
|  | **Project ID** | **Citations and document type** | **Country** | **Objectives** | **Brief Description of Agricultural and other Intervention Activities** | **Evaluation Design if available** | **Summary of dietary findings / outcomes** |
|  | B01 | (1) - Evaluation  (2) – Evaluation  (3) – Evaluation  (4) – Evaluation  (5) – Training manual  (6) - Project Approach | Bangladesh | Project aimed to improve household vegetable consumption through improved training of women in home gardens and nutrition | Introduced new nutrient rich vegetable varieties  Participants received one day intensive training on nutrition and garden establishment | Non randomized, controlled pre/post study  Control: n=103 women  Intervention: n=479 women | Vegetable diversity index significanly higher in intervention group at endline but no other diet diversity indicators assessed |
|  | B02 | (7) – Case study | Bangladesh | Project aimed to improve micronutrient intakes of women and children through disssemination of carp-mola polyculture technology, enhanced production of VAFV and nutrition SBC | Promoted increased consumption of micronutrient-rich small fish, FV, essential nutrition and essential hygiene actions  “practical” cooking classes. Addressed gender norms and attitudes in relation to food purchase, intra-household food  distribution and work load | Not reported | Increased fish consumption; but no diet diversity outcomes reported |
|  | B03 | (8) – Evaluation | Bangladesh | The project aimed to reduce vitamin A deficiency among women and children (6-59 months) in rural Bangladesh by increasing/improving availability and consumption of fruits and vegetables | - Establish community village nurseries to promote fruit and vegetable growth - Establish homestead gardens for year-round vegetable production - Provide nutrition education | Pre-post study (no control group) | Increased VA intakes from FV among women and children but no other dietary diversity outcomes reported |
|  | B05 | (9) – Evaluation | Bangladesh | Project aims to support development of small scale poultry value chains to improve women’s income and nutrition of women and children | Loans and technical assistance for different poultry enterprises given through women’s groups | Cross sectional comparative study  35 HH from adopting village;  25 HH from non-adopting village | No differeces in consumption of egg, poultry, fish, rice, vegtables  No diet diversity outcomes reported |
|  | B08 | (10) – Training description  (11) – Study protocol  (12) - Evaluation | Bangladesh | The project aimed to increase household level access to nutrient-dense food, including poultry and eggs, with the ultimate goal of increasing height-for-age of children during the first 1000 days | - Village Model Farmers (VMFs) selected and supported by the project to establish farms and support community members with homestead food production - Seeds provided to VMFs to grow and distribute - Nutrition training and at-home counseling monthly or bimonthly - Courtyard sessions on MIYCN targetted at mothers, husbands and grandmothers - Cooking demonstrations with women twice a year | Cluster randomized, controlled longitudinal cohort study  Intervention: n= 48 clusters; nBL: 1337 mother child pairs  Control: n= 48 clusters with 1368 mother child pairs.  Loss to follow up < 5% in both groups | Diet diversity outcomes for women / children not available. Egg consumption increased significantly among women and children |
|  | B10* | (13) – Policy Brief; (14) – Baseline study  (15) – Gender study  (16) – Evaluation report;  (17) – Outcomes brochure | Bangladesh | The Agriculture, Nutrition and Gender Linkages (ANGeL) pilot project aims to identify actions and investments in agriculture that can leverage agricultural growth to improve nutrition, and to enhance women’s empowerment in Bangladesh | Three primary activities provided in different combintations   - Agricultural training with emphass on production of fruits and vegetables; pulses; oilseeds; and poultry, dairy, fish, livestock - Nutrition social and behavior change targetting men and women - Gender sensitization | Cluster RCT with 5 arms + a control arm  T1: Nutrition BCC by government extension agents  T2: Nutrition BCC by ANGeL trained community nutrition workers  T3: Agricultural Production  T4: Nutrition BCC by government extension + Agricultural Production  T5: Nutrition BCC by government extension + Agricultural Production + Gender Sensitization  Control: no activities  Baseline Sample:  **Intervention Groups**: 25 blocks allocated to each treatment arm (125 blocks) with 625 households selected per block for a total of 3,125 intervention households  **Control**: 875 farm households across 35 blocks  26 households lost to follow up and equally distributed across arms. | DDS-C: “there was a statistically significant impact on child-level dietary diversity in arms T2 and T4. It was higher in T3 and T5 as well, but not statistically significant” |
|  | BF01* | (18) – Evaluation  (19) – Evaluation  (20) – Evaluation  (21) – Sustainability study  (22) – Mediation analysis  (23)– Process evaluation  (24) – Lessons learned report  (25) – Communications strategy  (26) – Project experience documentation  (27) – Formative study results | Burkina Faso | The project aimed to improve nutritional status of women and children and increase women’s empowerment through agricultural production | - Created village model farms as training sites for women - Provided inputs such as seeds, agricultural capital, and dry-season wells to VMFs - Women replicated farming activities at home - Women were visited by either frontline workers or older women leaders at home twice a month for counseling on nutrition behaviors | Cluster RCT  **(Intervention [health workers]:**  Baseline – 514 households  Follow-up – 441 households  **Intervention [older women leaders]:**  Baseline – 512 households  Follow-up – 443 households  **Control**  Baseline – 741 households  Follow-up – 597 households**)** | No significant impact on MDD-C, WDD scores (p<0.05) or HDDS |
|  | BF03* | (28) – Study design  (29) – Impact evaluation  (30) – Formative study  (31) – Program guide  (32) – Program guide  (33) – Evaluation  (34, 35) -Evaluation | Burkina Faso | Soutenir l’Exploitation Familiale pour Lancer l’Élevage des Volailles et Valoriser l’Économie Rurale (SELEVER) aims to leverage poultry value chain enhancement to improve household income, women’s empowerment and diets and nutritional status of women and children, | Intervention group received   - Improved access to poultry value chain services such as vaccinations, flock management training, financing / credit and inputs - BCC on nutrition and health - Community level sensitization on women’s economic empowerment and gender equity - An additional arm received the intervention activities + enhanced WASH | cRCT with repeat cross sectional surveys at baseline and endline  Control = 30 communities (nBL=507 CU5, 339 CG; nEL = 451 CU5; 326 CG)  NSA = 30 communtnies (nBL=473 CU5, 343 CG; nEL=450 CU5, 328 CG))  NSA+WASH = 30 communities (nBL= 488 CU5, 302 CG; nEL=414 CU5, 302 CG) | No statistically significant impacts on WDD scores or women’s micronutrient adequacy  No statistically significant impacts on DDS-C, MDD-C or WDD |
|  | Bo01 | (36) – Evaluation | Bolivia | Project aimed to increase poultry production. egg consumption, and subsequent micronutrient  and fat intake. | - Inputs for coop construction - Ten vaccinated chicks - 4-7 nutrition workshops per community provided training on family nutrition, egg consumption and optimal breastfeeding practices - 3-6 workshops on poultry feeding, breeding, disease - and pest management. | Pre-post, nonrandomized, noncontrolled study from 2013-2015 with rolling entry of 16 communities over project period  Of 430 households in 16 communities, 329 participated in project activities. Diet surveys conducted in random subsample  Baseline dietary surveys: 94 HH (65 children 6m-5y; 127 adult females; 110 adult males)  Endline dietary surveys: 27 HH (19 children 6m-5y; 29 adult women; 29 adult men) | No diet diversity outcomes reported;  Significantly increased egg consumption among all groups from baseline to endline |
|  | C01* | (37) – Evaluation  (38) – Process evaluation | Cambodia | The goals of the HFP program are to increase the diversity, year-round production and consumption of FV and ASF, increase income, and improve health and nutrition outcomes in participating women and children | village model farms  Agriculture training and inputs (seeds, seedlings, saplings, poultry, and animals)  Nutrition education | Nonrandomized, Pre – post, repeat cross sectional  Controls N=200; Intervention n=300 at both time points | Higher HDD among intervention HH at endline (p<0.05); greater increase in HDD among intervention relative to control  No effect of intervention on DDS-C or WDD indicators |
|  | C02* | (39) – Summary paper  (40) – Evaluation  (41) -Evaluation | Cambodia | The project aimed to contribute to reducing malnutrition in Cambodia by improving access, availability, and utilization of nutritious foods at the household level | - Farmers were grouped into Farmer Field Schools and trained on agricultural techniques and family nutrition - Farmer Business Schools that link farmers with markets - Agricultural fairs - Nutrition education, cooking demonstrations, and educational nutrition posters | Cluster RCT  **(Intervention:**  nBL: 508 mother-child pair; nEL: 524 mother-child pairs  **Control**  eBL: 233 mother-child pairs; nEL: 397 mother-child pairs | Significantly increased DDS-C ad MDD-C among children 6-23 months (both p<0.05)  No significant effects on HDD; Did not measure WDD |
|  | C03 | (42) – Evaluation  (43) – Evaluation | Cambodia | Fish on Farms aimed to evaluate the impact of an EHFP programme, with or without fishponds, on dietary intakes and nutritional status of women and young children. | VMF  Agricultural training and inputs (e.g., seeds, saplings, fish fingerlings, pond materials)  Small group and one on one education  Cooking demonstrations | cRCT  90 villages (clusters)  randomly allocated to one of three arms: (a) homestead gardens only; (b) home gardens plus fishponds or (c) control (no intervention). Ten HH per cluster then randomly selected for a total of 900 households recruited for evaluation (300 HH / arm) | “intervention households had higher food production, greater income from sale of surplus products, higher  household food security, dietary diversity, and  significantly higher intakes of key micronutrients in  comparison to the control” – (No data or p values provided) |
|  | C04 | (44) – economic evaluation  (45) – protocol | Cambodia | Family Farms for the Future aims to scale the Fish on Farms project to four regions, applying enhanced homestead food production (EHFP) to improve food security, women’s empowerment and nutritional status of subsistence farming households | VMF  Agricultural training and inputs (e.g., seeds, saplings, fish fingerlings, chicks)  Two sessions on i) IYCF and maternal nutrition following ENAs and WASH  Monthly gender equity training (7 sessions)  Women’s business and entrepreneurial training | pragmatic, delayed-intervention, cRCT (*Surveillance Trial*)  Of 232 participating villages (4642HH); 84 villages randomly sampled for surveillance trial with 318 HH from intervention 334HH from control villages | Children 6-59 mo: reduction in the prevalence of inadequate zinc and VA intake (no p value provided);  Women: no impacts on adequacy of zinc, VA or iron intake;  No diet diversity outcomes reported |
|  | Ch01 | (46) – pilot w evaluation | China | The objective of this project was to increase protein in the diet of people living with HIV/AIDS (60% women) by planting and consuming soybean. | - Participants were trained on soybean production, nutrition, and cooking and consumption methods - Participants received follow-up visits to ensure/encourage soybean consumption | Endline survey with 51 HH (no baseline) | diet diversity outcomes not reported |
|  | Co01 | (47) – Evaluation | Colombia | Project aimed to improve production and consumption of more nutritious yellow potato varieties | - Participatory selection of cultivars with communities - Development of Family Farmer Community Schools - Participatory education on linkages between agriculture and nutrition - Establishment of home gardens - Development and Implementation of community action plan - Rural entrepreneur groups - Leadership Schools in Food Sovereignity, food security and nutrition | No evaluation data available | Diet diversity or other dietary data not available |
|  | D01* | (48) – Evaluation  (49) – Operations research  (50) -Training manual  (51) – monitoring tool  (52) – Formative study  (53) – Formative study  (54) – preliminary evaluation presentation  (55) – Training guide | Democratic Republic of Congo | Jenga Jamaa II  aimed to empower women, increase income and improve food security and child nutrition through a multi-component nutrition sensitive intervention | A1: Farmer Field Schools received biweekly hands-on education on  farming practices, post-harvest natural resource management,  and business skills  A2: Farmer2Farmer engaged FFS beneficiaries to train others  A3: Child nutrition group received supports for home garden; Care groups for child nutrition and health, monthly food rations and health systems supports (no WE activities)  A4: WE held weekly WE groups, provided starter kits for income generating activities, goats and stoves and established women’s Savings and Credit groups | Quasi-experimental matched cohort design. Communities allocated to either individual intervention components or combinations. Participating women in each community matched to nonparticipating woman from same community    Control: n=265 HH  A1: n=206  A2: n=215  A3: n=393  A4: n=234 | Significantly increased DDS-C, MDD-C and % achieving MAD in A3 compared to control; significantly increased MDD-C in A1 and A3 relative to control |
|  | E01 | (56) – Case Study | Ethiopia | The project aimed to increase family welfare by improving productivity of goats managed by low-income women | - Provided 2 local female goats to women and extension support for improved goat breeds - Women organized into savings groups for managing goat credit disbursements and start small businesses - Small-scale backyard poultry program - Women trained on nutrition, VA rich foods, and business skills - Provided seeds, agricultural materials, poultry stock etc - Annual nutrition days | Pre-post analysis of 39 intervention HH | Data suggest increased child / household milk intakes but no p-values provided; No diet diversity outcomes reported |
|  | E02* | (57) – Evaluation  (58) – Evaluation | Ethiopia | Alive and Thrive: To improve infant and young child feeding practices in Amhara, Ethiopia, supporting the government’s National Nutrition Plan | - Health extension workers and volunteers provide nutrition education to mothers at health centers and via home visits - Agriculture extension workers provide support on establishing and maintaining home gardens and poultry - Mass media campaigns promote IYCF practices through radio dramas - Community conversations and church sermons target IYCF messages to the entire community | cRCT evaluation design with cross-sectional surveys at baseline (I=1328; C=1318 ) and endline (I=1360; C=1360). | No significant impacts on MDD-C (DiD p=0.08); WDD not assessed. |
|  | E03 | (59) – Study Protocol; (60) – Qualitative study  (61) -Evaluation | Ethiopia | The Sustainable Undernutrition Reduction (SURE) project aimed to increase dietary diversity and reduce stunting among young children through multi-sectoral collaboration, integrating agriculture activities into existing growth monitoring, nutrition and health activities | - Joint counseling of mother-father pairs on both agriculture and child nutrition by health and agriculture extension workers - Community dialogues of 30 households in men’s and women’s groups led by agriculture and health extension workers, respectively - Cooking demonstrations for network of 30 HHs/group led jointly by health and agriculture extension workers - Agriculture extension workers set up community gardens and conduct training on production of nutritious foods - Lowest income HHs provided one-time input of seeds and poultry for home gardens - Radio messages to promote IYCF | community based comparative cross-sectional endline study in program covered (n=416 HH) and noncovered (n=416) communities | Higher proportion of children in intervention area achieved adequate diet diversity |
|  | E05* | (62) – Evaluation(63) | Ethiopia | The project aimed to increase productivity of smallholder farmers, empower women farmers, and improve income through poultry rearing | - Households were provided with 25 chicks and basic advice on maintenance - Households were provided with fruit and vegetable seeds for home gardening. - Behavior change specialists conducted home visits and counselled women (and sometimes their husbands) on child feeding, finance management, and WASH | Cluster RCT  **Baseline (childer 0-23yo at baseline):**  Control – 328 children  Poultry only – 287 children  Poultry + nutrition SBC – 272 children  **Endline:**  Control – 311 children  Poultry only – 263 children  Poultry+ nutrition SBC – 255 children | Increased DDS-C among the poultry + nutrition SBC intervention group compared to the poultry only intervention group (p<0.05) but no significant effect when compared to the control group (p≥0.10) |
|  | E06* | (64-66) Evaluations | Ethiopia | Aimed to assess impacts of a child-owned poultry-nutrition intervention on egg intake, growth and development among children 6-18m | FLW capacity strengthening trainings  Chicken and Caging Gift Ceremony model (CCGC) led by religious leaders  SBCC focused on demonstrating how to prepare eggs and nutrition / WASH education focused on eggs, poultry production and environmental sanitation | cRCT with two communities selected and randomized to intervention (127 HH) or control (126 HH) | Intervention significantly increased DDS-C, MDD-C, consumption of eggs and VA rich FV; women’s diets not assessed |
|  | G02 | (67) – Baseline study  (68) - Evaluation  (69) – Baseline report  (70) – Baseline study (secondary analysis)  Phone call with project team member | Ghana | The project aimed to improve maternal and child nutrition and alleviate poverty for smallholder farm households using farm intensification technologies that sustainabliy increase production of nutritious foods | - Promotion of improved agricultural techniques and improved seeds/cultivars - Community cooking demonstrations and group cooking activities - Nutrition education to households | No evaluation document available | No outcomes reported |
|  | G03* | (71) – Presentation (72) – Evaluation | Ghana | Project aimed to “improve children's diets through increased home production of  nutrient‐rich foods and improved child‐feeding knowledge, income, and empowerment that would encourage purchases of nutrient‐rich food from markets” | Integrated package of agricultural inputs and training with nutrition education   - Agriculutre components included ( 1) Poultry for egg production;( 2 ) Home gardens as well - Group education on child diets with emphasis on consumption of eggs, green leafy vegetables, and orange‐fleshed sweet potatoes. - Community education including (a) food demonstration sessions (b) mother‐to‐mother support groups (c) enhanced community‐based growth monitoring and promotion, and (d) community‐wide discussions on gender and diversity. | Cluster RCT  **Intervention**: 19 communities / 194 mother-infant pairs enrolled at baseline of which 120 completed endline  **Control**: 20 communities / 122 mother-infant pairs enrolled at baseline of which 100 completed endline | Significantly increased egg consumption and MDD-C relative to controls |
|  | G05 | (73) – Program Description  (74) – Willingness to Pay Study | Ghana | The project aimed to improve the lives of farmers and increase pride in their work by providing agriculture and nutrition education and advice through a phone service | - Automated SMS messages on local weather and market price information sent to farmers multiple times a week - Automated voice messages on agriculture and nutrition customized based on the farmer’s language, crop of preference, location sent multiple times a week. - Farmers have free access to live phone support from agriculture experts - Farmers can call peers enrolled in the project for free to foster peer learning | Not described | “Women were more likely  to report changes in their nutritional practices,  including using new recipes and changing the  variety of food eaten” No diet diversity outcomes reported |
|  | Gu01Ŧ | (75) - Evaluation | Guatemala | Pilot study aimed to evaluate the effects of nutrition intervention alone or in combination with home garden inputs and training on food security, maternal and child diet diversity | Standard of care nutrition (SOCN) intervention: food rations, multiple micronutrient powders, five group nutrition classes, six once monthly home visits with counseling  SOCN+Agriculture intervention: gardening inputs, monthly home based garden training visits for 8m; 8 group / individual gardening classes | Pre-post pilot study in two communities, one each allocated to intervention or control with 70 HH selected per arm | No statistically significant impact on CDD, WDD |
|  | I02 | (76) – Evaluation  (77) – Formative study  (78) – Policy brief  (79) – project video  Email communication with project team | India | The project aimed to address household diet diversity through perennial kitchen gardens | - Perennial trees and green leafy vegetables provided to households for backyard gardening - Community interactions and home-visits for nutrition education - Community cooking demonstrations for women and school-children - Households encouraged to distribute excess seeds to neighbors | Pre-post repeat cros sectinal study in intervention communities  nBL=396 HH  nEL=362 HH | No significant increase in child consumption of green leafy vegetatbles; diet diversity outcomes not reported |
|  | I04* | (80) – Protocol for cost-effectiveness study  (81) – Impact pathway analysis  (82) – Feasibility study  (83) – Feasibility study  (84) – Program rationale  (85) – Formative study report  (86) – Evaluation  (87, 88) - evaluations | India | The Upscaling Participatory Videos ad Action for Agriculture and Nutrition (UPAVAN) project aimed to improve  maternal and child diets and nutrition through the dissemination  of locally developed videos, women’s group meetings and  follow-up  home visits | Four arms:  Control: standard government provided agriculture and health services  AGRI: twice monthly participatory NSA videos with community follow up visits and FLW training  AGRI-NUT: Once monthly participatory NSA videos; once monthly nutrition specific videos; follow up community visits; FLW training  AGRI-NUT-PLA: Once monthly participatory NSA videos; once monthly participatory meetings or nutrition specific videos; follow up community visits; FLW training  Participatory videos emphasized peer-to-peer learning, peer support, building women’s confidence, problem solving, and collective action | cRCT with repeat cross sectional surveys at baseline and endline  148 **clusters randomly allocated to 4 arms (37 clusters per arm).**  Control: BLn=1069  AGRI: nBL = 1118;  AGRI-NUT: nBL = 1104  AGRI-NUT-PLA: nBL =1189 | AGRI-NUT and AGRI-NUT-PLA incrased MDD-C relative to control;  AGRI and AGRI-NUT-PLA increased WDD relative to control |
|  | I05 | (89) – Evaluation | India | ‘Overcoming Poverty in Coconut Growing Communities: Coconut Genetic Resources for Sustainable Livelihoods in India’ aimed to improve food and nutritional security and income of the family members of the small and marginal coconut holdings | - Agriculture - Intercropping, nutrition gardening, livestock integration, product diversification - Extension - provision of inputs and appropriate technologies, village level equipment and capacity strengthening - Income generation activities - Nutrition education emphasizing utilization of available foods and production of high value products like baby food and other nutritional foods from locally available raw materials | Time series, pre-post without control group  150 Households from 3 communities (n=50 / community) | “significant improvements in the food and nutritional security, in terms of frequency and quantity of consumption as well as [women and children’s] diet diversity”; however no p values provided. Methods used to assess dietary diversity unclear; increased consumption of milk, FV in adults and children and egg in children |
|  | I06 | (90) - Evaluation  (91) – Annual Report | India | The project aimed to improve access to nutritious food and conserve groundwater by motivating farmers to divert parts of their land for planting nutritious crops | - Households taught farm-based technologies and provided inputs of nutritious and improved seeds - Nutrition education provided in central locations and at the health centers - Cooking demonstrations held for mothers and infants - Open air digital communication for larger community - Educational pamphlets provided to mothers | Pre-post intervention design without control  142 mother- child pairs | No diet diversity outcomes of interest reported;  Egg consumption increased but no tests of statistical significance performed |
|  | I07 | (92) – Baseline report  (93) – Adult learning process  (94) –Implementation guide  (95) – Training manual  (96) – Program report  (97) – Impact brief | India | The *Community Hunger Fighters Programme* aimed to enhance production of cereals and pulses, increase on-farm crop diversity to improve dietary diversity and promote vegetable cultivation of naturally biofortified FV via HH and community gardens | - Nutrition literacy program following adult education and community empowerment and critical reflectioin principles - Created “loose network” of community resource persons for nutrition literacy known as community hunger fighters - Community identified capacity building and knowledge sharing needs and activities on nutrition and agriculture - Street theatre, songs, visuals for mobilization, engagement and dialogue | Pre-post intervention design without control  374 households across 2 communities | Only household level dietary data reported |
|  | K01* | (98) – Evaluation;  (99) – Nutrition Education Protocol  (100) – Draft implementation manual  (101) - Cooking Demonstration protocol | Kenya | Aimed to assess “the effectiveness of a community‐based participatory approach in increasing micronutrient adequacy of diets of women and young children through agricultural  activities and nutrition education” | - Participatory workshops engaged communities to autonomously identify and plan agricultural activities to improve nutrition including - Development of community action plans on nutrition - Identification of poultry rearing and kitchen gardens as main agricluture activities - Community contract signing - Education on household diet diversity, complementary feeding, nutriiton in pregnancy | **Quasi-experimental**  **Intervention**: 5 sublocations with 167 mother – child pairs at baseline and 168 mother-child pairs at endline  **Control**: 5 sublocations with 165 mother-child pairs at baseline and 165 mother child pairs at endline | Significantly increased DDS-C, MDD-C and mean adquacy ratio compared to control (both p DiD <0.001) significant increases in child milk meat, legumes intake; No significant impact on womens diet diversity, nutrient adequacy; only milk intakes increased among women |
|  | K03* | (102) – Program design description  (103) – Evaluation  (104) -  (105) – Operations research report  (106) – Operations research results  (107) – Program brochure  (108) (109) | Kenya | Mama SASHA aimed to increase food security, vitamin A intakes and diet diversity of women and young children through increased production and consumption of biofortified OFSP | - Improved nutrition education and vouchers for OFSP vines offered to pregnant women attending antenatal care services - Community based mothers groups for PLW focused on nutrition education, recipes and cooking demos and OFSP agriculture - PLW redeemed vouchers for vines from model farmers who hosted OFSP extension activities - Commuity extension agents conducted home visits to support OFSP production | Quasi experimental, pre – post cross sectional surveys in control and intervention sites with nested longitudinal cohort study  505 mother child pairs in cohort study  Baseline cross sectional survey (nI =1388; nC=1373)  Endline cross sectional survey  n-I = 1163; n-C = 1108 | Significantly increased DDS-C and DDS-W; significantly increased VA intakes and consumption of OFSP among women and children |
|  | K04* | (110) - Evaluation  (111) – Study protocol | Kenya | This project aimed to assess the effectiveness of a bundle of basic agriculture, nutrition-specific, and nutrition-sensitive  interventions on child diets and growth compared to basic agriculture interventions. | Agricultultural inputs and biweekly agricultural training focused on home gardens (intervention and control)  Intervention group received additional inputs including micronutrient powders (MNP) for children 6-25mo; monthly soap distribution, ORS+zinc; chlorine water treatment solution every 6 months; 8 laying hens at enrollment and at 1 year follow up  Monthly nutrition and WASH trainings | Cluster randomized controlled cohort study  126 study communities  1000 children 6-35m recruited per arm; 818 and 745 retained through 2 y in intervention and control groups respectively | MDD-C significantly higher in intervention;  Significantly greater number of eggs consumed by children in intervention arm |
|  | K05 | (112) - Evaluation  (113)- Evaluation | Kenya | This study aimed to assess the effects of single-component vs multi-component nutrition education on HH production of OFSP and OFSP consumption by preschoolers | OFSP promotion – vine distribution and cooking demonstrations (all)  Arm 1 (preschooler arm - PA): OFSP-branded exercise books and posters used by ECD teachers with preschool children  Arm 2 (caregiver arm- CA): dissemination of daily OFSP and nutrition oriented text messages through their mobile phones (30d)  Arm 3: (Multicomponent Arm, MCA): concurrently providing OFSP exercise books and posters via preschoolers + mobile phone messages to caregivers | cRCT  12 communities randomized to each of 3 treatment arms (4/arm) and 3 villages in the  control group  431 HH completed baseline surveys (n=76 PA; 82 MCA; 132 CA; 100 control) and 360 completed follow up survey 7 months later (n= 68 PA; 77 MCA; 121 CA; 94 control) | No dietary diversity outcomes; data on OFSP consumption not reported |
|  | K06 | (108) - Evaluation (109) – Evaluation | Kenya | The Scaling Up Sweetpotato Through Agriculture and Nutrition (SUSTAIN) aimed to improve food security and vitamin A status through scaled production of biofortified OFSP. | Health talks and cooking demonstrations at ANC / PNC and mother clubs  One on one nutrition counseling; network of stakeholders  Health talks at community based agricultural extension events  Radio talk show focused on MCN, VA and agriculture  Agriculture extension focused on OFSP production methods, handling and storage, pest management and vine propogation through home based and community based extension events | Midline survey with 585 participants; no control; unclear if baseline was conducted | No dietary diversity indicators reported |
|  | M01* | (114) - Evaluation  (115) – Evaluation  (116, 117) – economic evaluations | Malawi | Improve production diversity, maternal knowledge on nutrition and child feeding, and children’s diets and anthropometric measures | - Train and support community members to establish and maintain community gardens at community based childcare centers (CBCCs) - Train and support community members to establish home gardens to support meal preparation at CBCC - Train caregivers and CBCC staff on child nutrition and nutritious meal preparation | Cluster RCT  (**Intervention**:  Baseline – 637 children 3-6 years old; 167 children 6-24 months old  Endline - 533 children 3-6 years old; 115 children 6-24 months old  **Control**:  Baseline – 619 children 3-6 years old; 152 children 6-24 months old  Endline - 558 children 3-6 years old; 132 children 6-24 months old) | “positive effect on household dietary diversity” (no data reported)  Increased DDS-C among children 3-6 yo (p<0.001);  data on younger children not reported |
|  | M02 | (118) – Evaluation  (119) – Case study  (120) – Qualitative study  (121) – Qualitative study | Malawi | Aimed to improve child nutritional status in farming households through a participatory approach for legume intercropping | - Promotion of legume intercrops that previous research showed would be accepted in the community - Community activities such as recipe days and crop residue promotion days to address women’s role in household and transfer skills on diverse diets - Intergenerational discussion groups meet monthly to discuss gender and nutrition issues | “prospective quasi-experimental study comparing baseline and follow-up data in ‘intervention’ villages with matched subjects in ‘comparison’ villages”  “Over a 6-year period, nine surveys were conducted, taking 3838 height andweight measures of children under the age of 3 years (n=566)” | No women / child diet diversity outcomes reported |
|  | M03 | (122) – Evaluation  (123) – Evaluation | Malawi | The project aimed to improve food security and dietary diversity through participatory agroecology practices in vulnerable family farming households. | - Farmers experiment with their chosen methods of agroecological techniques to increase soil fertility, productivity, and diversity. Farmers are encouraged to share knowledge with each other. - Livestock managed at the community level then offspring distributed to participating farmers - Monthly field visits were provided to farmers by the project team - Seed bank established and farmers assisted with seeds and training at experimentation farms - Monthly facilitated group discussions on social norms, nutrition, and gender | Pre-post study (no control group)  Baseline: 306 households  Endline: 352 households | No statistically significant change on HDDS |
|  | M06* | (124) – Process review  (125) – Evaluation | Malawi | This project aimed to test whether community nutrition education + food security strategies improved child diet diversity to a greater extent than food security strategies alone | - Participatory nutrition education (NE) curriculum encouraged HH to include more local and seasonal food with emphasis on vegetables, fruits, pulses and animal source foods in diets of children 6-23 months - Food security activities (FSA) included Farmer Field Schools, Junior Farmer Field and Life Schools, and farmer field days - Participants also received inputs including seeds, fertilizer, fruit tree seedlings and livestock | Cluster RCT  **Intervention** (NE+ FSA): baseline = 419 HH; endline = 493 HH  **Control** (FSA only): baseline = 420 HH; endline = 466 HH | Significantly improved DDS-C (pDiD=0.01) MDD-C (pDiD=0.01) and MAD (pDiD=0.02)  Significant increases in egg and groundnut consumption by children in intervention area compared to control area, consumption of eggs increased by 10 percentage points (p<0.01) |
|  | Mo01* | (126) – Evaluation  (127) – Evaluation | Mozambique | The project aimed to reduce vitamin A deficiency through agriculture, nutrition, and marketing intervention components | - Orange fleshed sweet potato (OFSP) vines were distributed to farmers annually along with training on growing techniques - Extension meetings – conducted after vine distribution to discuss growing, storing, and consuming OFSP - Volunteer promoters were selected from each farmer group to reinforce messages from extension meetings. Promoters also visit farmer fields to provide support |  | Improved DDS-C for children <5 years (p<0.05) . Also reported improved child vitamin A and OFSP intakes |
|  | Mo02* | (128) – Evaluation | Mozambique | The *Towards Sustainable Nutrition*  *Improvement Project (TSNI)* tested  “whether an integrated agriculture–nutrition project with a significant market development component  using OFSP as the key entry point could [improve] vitamin A intake and serum retinol concentrations in children under 5 y”. | The 3 intervention components included agriculture extension promoting OFPS, demand creation/behavior change, and marketing.  The intervention and control groups both received public awareness campaign on benefits of OFSP | quasiexperimental, prospective, controlled, and longitudinal study    data available for 53 farmer groups (323 men and 718 women; nI 465; nC 234) | Significantly improved DDS-C and MDD-C as well as consumption of OFSP and VA |
|  | N02 | (129) – Process evaluation  (130) – Evaluation; (131) – Women’s empowerment study; (132) – SBC strategy; (133) – Technical brief; (134) – Implementation Brief; (135) – Egg promotion campaign strategy; (136) – Formative study  (137) – Midline evaluation | Nepal | This homestead food production project aimed to improve maternal and child nutrition, increase use of nutrition and health services, and strengthen coordination among nutrition actors | - Households were provided vegetable seeds, chicks, and training on homestead gardening - Village model farms established for demonstration - Frontline counseled mothers at home - Mothers’ groups established - Listening groups used to listen to and discuss radio messages - Self-monitoring household tool used to improve WASH behaviors - Nutrition assessment and counseling services provided at health centers to prevent and treat malnutrition | Pre-post study (no control group, data from program monitoring)  2101 mothers; 994 children 6-23mo (sample size at various timepoints not specified) | CDD and WDD from 7 day recall significantly higher in intervention compared to control, except in rainy season. |
|  | N03 | Evaluation - (138) | Nepal | The Market Access for Rural Development (MARD) project aimed to 1) increase household income by increasing production and sale of cash crops (primary objective) and 2) Improve nutritional status of women and children <5 years old | - Nutrition demonstration households established to serve as model kitchen gardens - High economic crop value demonstration sites established - The project provided seeds and training - Factsheets with nutrition facts distributed to households - Kitchen gardens established to plant high-economic value vitamine A crops | Single cross-sectional survey at endline with 430 participating households and 389 control households from nonproject communities | Higher reported HH consumption of DGLV, VAFV but not ASF among intervention HH. CDD, WDD not reported |
|  | N04 | (139) - Evaluation | Nepal | This Heifer International Project sought to test whether the addition of community development activities to nutrition sensitive agriculture improved child diets and growth to a greater extent than nutritio sensitive agriculture alone. | (i) Full Package = community development activities focused on poverty alleviation, citizen empowerment and community  development, delivered via women’s groups; livestock training with emphasis on goats and poultry, provision of goat at end of training and concurrent nutrition education with emphasis on ASF consumption by infants and women vs.  (ii) livestock and nutrition education activities above but without community development (Partial Package); vs  (iii) no intervention (Control). | 3-armed longitudinal cRCT with five data collection points over 36m  Full – 290 HH  Partial – 366 HH  Control – 304 HH | Full intervention demonstrated significantly improved DDS-C compared to partial and control. No difference in change in DDS-C between partial and control. |
|  | N05* | (140) – Evaluation  (141) – Evaluation  (142) – Gender study  (143) – Formative study | Nepal | The project aims to improve maternal and child (0-23 months) dietary diversity and food security by increasing production and consumption of nutrient rich fruits/vegetables/ASFs through poultry rearing, home gardens and nutrition education. | - Establish model farms with trained village model farmers (VMFs) that provide training and technical support for community members - Provide inputs (seeds, chicks] to start model farms and for poultry rearing - Monthly meetings for nutrition education, cooking demonstrations, and routine health services | Cluster RCT  **(Intervention:**  Baseline – 1055 mother-child (12-48 months)  Follow-up – 1307 mother-child pairs  **Control**  Baseline – 1051 mother-child (12-48 months)  Follow-up – 1301 mother-child pairs) | MDD-C in the intervention group significantly higher than control group at follow-up (p<0.05) despite control having significantly higher MDD-C than intervention group at baseline (p<0.05) |
|  | R01* | (144) – baseline report  (145) – evaluation | Rwanda | Evaluate the added value of a nutrition SBCC intervention on child milk intake among HH in the government sponsored One Cow per Poor Family (Girinka) program | Controls: Girinka and nutrition standard of care activities  Interventions: CHW supervisors trained to train CHWs on how to use the SBCC materials and conduct household and community SBCC sessions. Sessions focused on promoting milk consumption (1c/day) among children >12m | cRCT | Increased child milk intakes but no statiscally significant impact on MDD-C |
|  | R02 | (146) - Evaluation | Rwanda | Evaluate added value of father engagement to SBCC to promote dairy consumption in One cow per poor farmer Girinka program | Controls: Girinka program activities + SBCC sessions  Intervention: Father focused approach including monthly group meetings for men, weekly text messages to men’s  mobile phones, and megaphone blasts.  Identification model fathers to serve as community change agents and group facilitators | Pre-post evaluation design without controls | Increased child consumption of ASF but no diet diversity outcomes reported. |
|  | S01 | (147) – Lessons learned (multi-country)  (148) - Sustainability Evaluation (Senegal only)  (149) -lessons learned and process indicators (multi-country) | Senegal | The Creating Homestead Agriculture for Nutrition and Gender Equity (CHANGE) project sought to improve the nutritional status of women and children under 5 years of age by encouraging women to produce vegetables  and eggs, improve nutrition and hygiene practices, and  support women’s empowerment. | The intervention  consisted of three main components: 1) horticulture and aviculture – including provision of two “microgarden” tables, a henhouse, chickens, inputs, training; 2) SBCC on nutrition and hygiene. Agriculture training and 3) women’s empowerment (not all communities in intervention arm received). Activities accomplished through monthly agriculture group sessions, monthly nutrition and hygiene SBCC sessions and home visits | Repeat cross sectional surveys during project (sample size not provided) and with 247 former participants 18 months after project end; semi-structured interviews with 20 women who were former participants. | Increased WDD over the project period but declined to baseline levels at the 18 months post project survey (no p values reported). CDD not reported; only child OFSP intakes increased over project period |
|  | SA01 | (150) – Summary  (151) - Evaluation | South Africa | This study aimed to assess whether integration of a homestead gardening program with growth monitoring improves intake of Vitamin A rich fruits and vegetables (VAFV) and serum retinol status of children 2-5y. | Demonstration gardens established at each home based growth monitoring center (*Isizinda)* and planted with VAFV.  Production and consumption of VAFV promoted at *Isindiza* by local nutrition monitors.  Cooking demonstrations and nutrition education conducted during grown monitoring sessions at *Isindiza* with emphasis on focused on the  relation between vitamin A and health, the identification of vitamin  A–rich foods, cooking methods, and the importance of a home  garden as source of vitamin A–rich foods. | Repeat cross sectional surveys at baseline and endline with one intervention village and one control village; nonrandomized.  Baseline: Of 129 children 2-5y recruited, 97 completed the baseline survey. Control: Of 85 children recruited, 48 completed the baseline survey.  Intervention village: of 127  children recruited, 108 completed the endline survey.  Control village: of 113 children recruited, 100 completed the endline survey | Intakes of VA rich FV significantly increased over the 20 months in the intervention village (p<0.01) but not in the control village. No diet diversity outcomes reported |
|  | SA02 | (152) – Evaluation | South Africa | The project aimed to decrease Vitamin A deficiency through increased production and consumption of β-carotene-rich vegetables, nutrition education, and community-based growth monitoring | - Health volunteers trained on growth monitoring and cultivation of β-carotene-rich vegetables. With demand creation for these vegetables, the goal was for health volunteers to earn an income from production - Monthly farmer forums supported by agriculture extension workers served as avenues for decision making and problem solving - Health volunteers held monthly growth monitoring sessions for children 1-5yo at community spaces to a) refer children with faltering growth to clinics b) hold cooking demonstrations c) provide nutrition education - Demonstration gardens for demonstrating cultivation and cooking of β-carotene-rich vegetables | Cross sectional endline in 209 participating and 223 nonparticipating households. No baseline data available | No diet diversity outcomes reported; increased consumption of VA rich FV. |
|  | SL01* | (153) – Evaluation | Sierra Leone | Project is designed to address food and nutrition insecurity among smallholder cocoa, cashew, and coffee farmers by promoting good agriculture and nutrition practices along the value chain | - Extension workers trained on agricultural techniques in established farmer field schools. These extension workers then train smallholder farmers - Inputs (improved seeds and agricultural materials) provided to farmers - Nutrition education provided to households - Gender sensitization activities | Quasi-experimental study  **Baseline –** 912 smallholder cashcrop farming households  **Follow-up –** 836 cashcrop farming households (cashcrop intervention only=251 HHs; nutrition education only=130 HHs; both interventions=196 HHs; control=236 HHs) | For the cashcrop + nutrition education intervention:  Increased DDS-C for children 6-59 months (p<0.05)  Increased WDD scores (p<0.05) |
|  | T01* | (154) – Spillover study  (155) – Study protocol  (156) – Midline evaluation  (157) - Evaluation | Tanzania | The project aims to improve dietary diversity and consumption of nutrient dense foods by engaging women in agricultural production | - Agriculture extension workers create farmer field schools (FFS) in one household/hamlet. Farmers attend sessions at the FFS every two weeks. - Women whose gardens were thriving served as model farmers and shared their experience and taught their peers best practices for home gardening - Farmers are provided agricultural inputs such as locally produced seeds - Nutrition counseling focused on infant and young child feeding with additional basic public health messages - Home visits by agriculture, livestock and health extension workers on rotation every two weeks | Cluster RCT  **Baseline** – 504 intervention, 502 control women  **Endline –** 452 intervention, 422 control women | Increased WDD scores (p<0.001) at one year but not at 3 years; nonsignificantly increased consumption of legumes, VA rich vegetables and fruits |
|  | T02 | (158) – Sustainability study | Tanzania | Assess long term (5y post) sustainability of a horticulture and nutrition project on maternal and child VA intakes and status | Homestead gardening training with emphasis on VAFV provided to households  Guava and pawpaw seedlings distributed to homes and school  Two-day nutrition education seminar for village women selected as health educators | Post test design completed 5y after intervention ended  HHs randomly selected from 5 intervention (n=125 HH) and 5 control (n=125 HH) villages to participate in sustainability survey | Significantly greater intakes of VA rich foods reported in previous 7 days among children in intervention communities. No diet diversity indicators reported. Women’s dietary data not reported |
|  | T03* | (159) – Evaluation  (160) – Curriculum development description | Tanzania | This agroecology focused project aimed to improve child nutrition, women’s empowerment and wellbeing, and sustainable agriculture | - Male and female mentor farmers selected in each village and trained on agroecological techniques, nutriton, gender, and social equity. They then train other farmers in the village - Mentor farmers invite community members to monthly meetings and conduct household visits quarterly - Intervention households are provided legume seeds for experimentation - Mentor farmers initiated other events based on need, including community wide discussions on gender | Cluster RCT  **Baseline –** 295 intervention, 296 control households  **Follow-up –** 255 intervention, 277 control households | Increased DDS-C for children 6-23 months (p<0.01)  Increased MDD-W (p<0.01)  No statistically significant change in MDD-C |
|  | T05* | (161) – Evaluation | Tanzania | Aimed to reduce malnutrition through diet diversification by promoting production and consumption of traditional African vegetables to increase nutrition security and household income | - Demand creation for traditional vegetables: cooking shows, road shows, sensitization campaigns in hospitals, schools, markets, and villages - Provision of English and Swahili fact-sheets with health and nutrition information to households with children under 5 years old and women of reproductive age | Quasi-experimental (propensity score matching method)  Total sample size of 500 households with children <5yo and women of reproductive age (same households at baseline and endline) – 258 intervention and 242 control households | Increased DDS-C for children <5 years (p<0.01) and increased WDD (p<0.001)  No statistically significant changes in HDDS |
|  | Th04* | (162) – Evaluation  (163) – Baseline study  (164) – Formative study  (165) – Process study | Thailand | The aim was to create long-term improvements in nutrition in the community by identifying local and practical agricultural solutions | - Community members trained on chicken coop construction and maintenance - Each household received 5 immunized egg-laying hens and 5 types of vegetable seeds - 1-hour community nutrition education sessions were provided, focused on infant and young child feeding - Monthly household visits to provide support/counsel on agricultural and IYCF techniques | Quasi-experimental - pre-post logitudinal field trial,, nonrandomized  **Baseline –** 125 intervention, 106 control children  **Endline** – 112 intervention, 93 control children | No statistically significant effect on DDS-C or MDD-C |
|  | TL01* | (166) – Conference paper  (167) – Program strategy  (168) – SBC strategy  (169) – Midline evaluation | Timor Leste | The *To’os ba Moris Di’ak* (Farming for Prosperity*)* Program (TOMAK) aimed to improve HH income, food security, rural market systems and nutrition of WRA and CU2 | establishment of farm demonstration plots, home gardens, and fish ponds, with farmer training and promotion activities;  facilitation of inputs and improved supply chains;  establishment of savings & loans (S&L) groups;  integration of nutrition SBC into existing farmers, mothers, church, and youth groups  Radio spots promote nutrition, poultry care, egg production and consumption  Community nutrition drama  theatre at community events, schools  Print media (recipe cards, posters, stickers, calendars) promoting poultry/ egg production and nutritious food consumption | Pre-post repeat cross sectional surveys. Household categorized based on self selection into different intervention activities: nutrition, S&L, farmer, multiple groups or no group (control). Differences at midline compared across groups | No significant differences seen between groups for MDD-C, MAD or food consumption scores; 63% of women in savings and loans groups achieved minimum diet diversity compared to 45% in the control group but no differences seen between other groups and control. |
|  | U01 | (170) – Evaluation | Uganda | The Reaching End Users project aimed to promote the production and consumption of OFSP at scale (>10,000 reached) to improve vitamin A intakes among children aged 6–35 mo and women . | - agricultural component included OFSP vine distribution and farmer groups training on improved production practices - The demand creation/ behavior change emphasized child and maternal health and nutrition topics targeted to women in farmer group households - Marketing campaign - a broader campaign for thegeneral public to raise awareness of the benefits of OSP as a source of vitamin A through community drama, field day events, and radio spots and programs | 3-armed cRCT trial of an intensive program (IP), a  reduced program (RP), which differed primarily in duration of exposure  to farmer group-level input and a control arm  84 farmer gropus with 35-35 members randomized; data collection on  Children 6-35m (n = 100 IP; n= 66 RP; n= 98 controls  Children 3-5y (n=205 IP; n= 131 RP; n= 208 control)  WRA (n=205 IP; n= 131 RP; n= 203 control) | Child / women’s diet diversity not reported. Intervention associated with significantly increased OFSP consumption and VA intakes. |
|  | V01 | (171) – Spillover study  (172) – Blog post | Vietnam | The project aimed to improve household food security and nutrition through homestead food production | - Village model farms (VMFs) were established and used as training and demonstration sites for households within that village. - VMFs and households were provided seeds, chicks, pigs, and composting tools. - Health workers conducted nutrition education sessions during household visits and in the clinic - Community events – health and nutrition festival days, cooking demonstrations, community radio talk show | Not described | “The vast majority of children in participating households had an adequately diverse diet including four or more food groups” no data or p values provided; |
|  | X01 | (173) – Lessons Learned Report | Multiple countries: Ethiopia, Kyrgyzstan, Nepal, Pakistan, and Peru | Aims to improve the nutritional status of people in mountain regions through financing and capacity building of microinterventions run by rural service providers focused on nutrition-sensitive agricultural programs | - Vegetable gardens established in schools and in households. Parents were trained to run and maintain the gardens - Low-cost green houses installed to enhance plant growth and income generation - In-person and radio-based awareness activities with messages on dietary diversity, agroecological production, consumption of nutritious foods - Livestock and fish interventions: fish farming, guinea pig production, poultry farming, goat milk promotion, beekeeping for income - Promotion of planting of fruit trees (and selling surplus in local market) - Training on value addition and conservation techniques - Note: combinations of interventions varied by country | Pre-post study (no counterfactual)  **Pre- and post-intervention**: 2300 women ages 15-49 that participated in the interventions across 5 countries completed food 24 hour recalls | Increased WDD scores (p<0.05) in Ethiopia, Pakistan, and Peru  No statistically significant changes in Kyrgistan |
|  | X04 | (174) – Project brief  (175) – Behavior Change strategy  (149) – Case study  (147) – Case study  (176) – Formative study  (177) – Facilitation guide  (178) – Formative study | Multiple countries: Burkina Faso, Senegal, Cotê d’Ivoire, Tanzania | The project aimed to improve the nutritional status of women and children through household food production and increased hygiene | - Households were provided with seeds and training, including demonstrations, on homestead gardening, with emphasis on production for consumption - Education on poultry rearing including a demonstration poultry pen in each sub-village, with emphasis on at-home consumption of eggs - Learning sessions on breastfeeding, complementary feeding, and maternal nutrition, targeting mothers, older siblings, and other members of the family | No diet quality outcomes of interest reported | No diet diversity outcomes reported |
|  | X05 | (179) – Evaluation | Multiple countries: Bangladesh, Cambodia, Nepal and the Philippines | The project aimed to increase consumption of fruits, vegetables, and animal source foods and improve dietary diversity of women and children through homestead food production and nutrition education | - Women’s groups of household producers and farmers were established and linked to health, agriculture, and market services - Village model farms (VMFs) established – VMFs received seeds and agricultrual training. They were then responsible for training and providing seeds to women’s groups in their area | No diet quality outcomes of interest reported | Increased HH production diversity; increased child vegetable and and egg consumption and women’s egg consumption; womens / children’s diet diversity data referenced but not reported |
|  | Z02 | (180) – Evaluation | Zambia | The aim was to improve the dietary diversity and nutrition of farming households through improved agricultural practices and increased production of nutritious crops | - Meetings with community stakeholders to discuss intervention activities - Households provided with vegetable and legume seeds - Nutrition education by community facilitators and extension workers to reinforce intervention activities | Quasi-experimental, endline only (no baseline)  348 intervention households and 194 non-intervention households | Higher HDDS among intervention households compared to control households at endline (p<0.01); individual dietary data not reported. |
|  | Z04* | (181) – Evaluation  (182) – Evaluation | Zambia | The project aimed to address child undernutrition through a multisectoral approach – agricultural diversification to increase access to nutritious food, promotion of gender equality and women’s empowerment, and nutrition BCC | - Provision of seeds and agricultural tools along with agricultural training - Provision of chickens and goats along with training on animal rearing - Women’s groups were established and smallholder model farmers (SMFs) selected. SMFs received agricultural training and inputs from project staff and government extension workers. SMFs pass on training and inputs to their women’s groups - Nutrition BCC provided in select women’s groups. Trained community health workers (CHWs) led BCC sessions during women’s group meetings. - SMFs and CHWs conducted home visits in between meetings | Cluster RCT (repeated cross-sectional evaluation surveys)  Analytic sample included children 6-24 months and their mothers  **Baseline:** 1524 (938 in treatment and 586 in control households)  **Endline:** 1343 (841 in treatment and 502 in control households) | No statistically significant changes in MDD-C, DDS-C, or WDD |
|  | Zi01 | (183) – Evaluation | Zimbabwe | The Livelihoods and Food Security Programme  (LFSP) aimed to improve food security and nutrition of smallholder farmers and rural communities | Nutrition education  Farm production diversity Commercialization | Cross sectional study, noncontrolled; Endline only  2814 households; 2,285 women and 506 children 6-23m | Negative binomial regression used to estimate independent effects of exposure to different program components on child / women’s diet diversity. No results presented for combination of nutrition SBC and agriculture |
| Abbreviations used in table: CDD – child diet diversity; DDS-C – diet diversity scores, children; FV – fruits and vegetables; HDD – household dietary diversity scores; HH- household; MDD-C minimum diet diversity, chidren; MAD – minimum acceptable diet;VA – vitamin A; WDD – women’s diet diversity; Ŧ not included in effectiveness ratio estimations because both groups received nutriiton SBC.  Note: some documents from included projects were not abstracted or used in analysis because they contained duplicate information, reported on non-diet outcomes, or did not contain information relevant for this specific analysis. | | | | | | | |

**Citations**

1. Baliki G, Brock T, Schreinemachers P, Uddin MN. Long-term behavioural impact of an integrated home garden intervention: evidence from Bangladesh. Food Security. 2019;11(6):1217-30. doi: 10.1007/s12571-019-00969-0. PubMed PMID: WOS:000501071800004.

2. Schreinemachers P, Patalagsa M, Islam MR, Uddin MN, Ahmad S, Biswas S, et al. The effect of women’s home gardens on vegetable production and consumption in Bangladesh. Food Sec. 2014;7:97-107. doi: <https://doi.org/10.1007/s12571-014-0408-7>.

3. Patalagsa MA, Schreinemachers P, Begum S, Begum S. Sowing seeds of empowerment: effect of women’s home garden training in Bangladesh. Agrculture & Food Security. 2015;4(24). doi: <https://doi.org/10.1186/s40066-015-0044-2>.

4. Schreinemachers P, Patalagsa MA, Uddin MN. Impact and cost-effectiveness of women’s training in home gardening and nutrition in Bangladesh. Journal of Development Effectiveness. 2016;8(4):473-88. doi: 10.1080/19439342.2016.1231704.

5. Homestread Vegetable Garden: Training Manual. n.d.

6. The World Vegetable Center’s Approach to Household Gardening for Nutrition. Shanhua, Taiwan: World Vegetable Center, 2016.

7. Castine SA, Bogard JR, Barman BK, Karim M, Hossain MM, Kunda M, et al. Homestead pond polyculture can improve access to nutritious small fish. Food Security. 2017;9(4):785-801. doi: 10.1007/s12571-017-0699-6. PubMed PMID: WOS:000406656300011.

8. Taher A, Talukder A, Sarkar NR, Bushamuka VN, Hall A, De Pee S, et al. Homestead gardening for combating vitamin A deficiency: The Helen Keller International, Bangladesh, experience2004. 68-74 p.

9. Nielsen H, Roos N, Thilsted SH. The Impact of Semi-Scavenging Poultry Production on the Consumption of Animal Source Foods by Women and Girls in Bangladesh. Journal of Nutrition. 2003;133(11 SUPPL. 2):4027S-30S. <http://www.embase.com/search/results?subaction=viewrecord&from=export&id=L37392587>.

10. HKI. HKI/FAARM Implementation Guideline sections in relation to trainings given. n.d.

11. Wendt AS, Sparling TM, Waid JL, Mueller AA, Gabrysch S. Food and agricultural approaches to reducing malnutrition (FAARM): Protocol for a cluster-randomised controlled trial to evaluate the impact of a Homestead Food Production programme on undernutrition in rural Bangladesh. BMJ Open. 2019;9(7). doi: 10.1136/bmjopen-2019-031037.

12. Lambrecht NJ, Waid JL, Wendt AS, Sobhan S, Kader A, Gabrysch S. Impact of a Homestead Food Production program on poultry rearing and egg consumption: A cluster-randomized controlled trial in Bangladesh. Matern Child Nutr. 2023;19(3):e13505. Epub 2023/03/25. doi: 10.1111/mcn.13505. PubMed PMID: 36961298; PubMed Central PMCID: PMC10262892.

13. Ahmed A, Ghostlaw J. Diversifying Rice-centric Agriculture and Diets: the Bangladesh Experience Agriculture for improved nutrition: Seizing the momentum. Wallingford, UK: International Food Policy Research Institute (IFPRI) and CABI; 2019.

14. Ahmed A, Ghostlaw J, Haque ML, Hossain NZ, Parvin A, Sufian FD, et al. Agriculture, Nutrition, and Gender Linkages (ANGeL) Baseline Study. Bangladesh: International Food policy Research Institute (IFPRI), 2017.

15. Quisumbing A, Ahmed A, Hoddinott J, Pereira A, Roy S. Designing for empowerment impact in agricultural development projects: Experimental evidence from the Agriculture, Nutrition, and Gender Linkages (ANGeL) project in Bangladesh. World Dev. 2021;146:105622. Epub 2021/10/05. doi: 10.1016/j.worlddev.2021.105622. PubMed PMID: 34602710; PubMed Central PMCID: PMC8350314.

16. Ahmed A, Hoddinott J, Menon P, Quisumbing AR, Roy S, Younus M. Agriculture, Nutrition, and Gender Linkages (ANGeL) evaluation results. 2018. doi: <https://doi.org/10.2499/p15738coll2.136540>.

17. IFPRI. Agriculture, Nutrition, and Gender Linkages (ANGeL). 2018.

18. Dillon A, Arsenault J, Olney D. Nutrient Production and Micronutrient Gaps: Evidence from an Agriculture-Nutrition Randomized Control Trial. American Journal of Agricultural Economics. 2019;101(3):732-52. doi: 10.1093/ajae/aay067. PubMed PMID: WOS:000480792300006.

19. Olney DK, Pedehombga A, Ruel MT, Dillon A. A 2-year integrated agriculture and nutrition and health behavior change communication program targeted to women in Burkina Faso reduces anemia, wasting, and diarrhea in children 3-12.9 months of age at baseline: a cluster-randomized controlled trial. J Nutr. 2015;145(6):1317-24. Epub 20150422. doi: 10.3945/jn.114.203539. PubMed PMID: 25904734.

20. Olney DK, Bliznashka L, Pedehombga A, Dillon A, Ruel MT, Heckert J. A 2-Year Integrated Agriculture and Nutrition Program Targeted to Mothers of Young Children in Burkina Faso Reduces Underweight among Mothers and Increases Their Empowerment: A Cluster-Randomized Controlled Trial. J Nutr. 2016;146(5):1109-17. Epub 20160413. doi: 10.3945/jn.115.224261. PubMed PMID: 27075910.

21. Dillon A, Bliznashka L, Olney D. Experimental evidence on post-program effects and spillovers from an agriculture-nutrition program. Econ Hum Biol. 2020;36:100820. doi: 10.1016/j.ehb.2019.100820. PubMed PMID: 31683110.

22. Heckert J, Olney DK, Ruel MT. Is women's empowerment a pathway to improving child nutrition outcomes in a nutrition-sensitive agriculture program?: Evidence from a randomized controlled trial in Burkina Faso. Soc Sci Med. 2019;233:93-102. Epub 20190516. doi: 10.1016/j.socscimed.2019.05.016. PubMed PMID: 31195195; PubMed Central PMCID: PMC6642337.

23. Nielsen J, Olney DK, Ouedraogo M, Pedehombga A, Rouamba H, Yago-Wienne F. Process evaluation improves delivery of a nutrition-sensitive agriculture programme in Burkina Faso. Maternal and Child Nutrition. 2018;14(3). doi: 10.1111/mcn.12573.

24. Olney DK, Dillon A, Ruel MT, Nielsen J. Lessons learned from the evaluation of Helen Keller International’s enhanced homestead food production program. Achieving a nutrition revolution for Africa: The road to healthier diets and optimal nutrition. Washington, DC2016. p. 67-81.

25. Rouamba H, Keith N. Stratégie de communication pour améliorer les pratiques en matière d’AEN dans le cadre du projet E-HFP/HKI District Sanitaire de Fada, Burkina Faso. Helen Keller International, n.d.

26. HKI. Capitalisation de l'experience du projet Enhanced Homestead Food Production HKI/Burkina Faso. . 2012.

27. HKI. Synthese des travaux sur l'auto-diagnostic communautaire dans les 30 villages d'intervention du project E-HFP. n.d.

28. Gelli A, Margolies A, Santacroce M, Sproule K, Theis S, Roschnik N, et al. Improving child nutrition and development through community-based childcare centres in Malawi - The NEEP-IE study: study protocol for a randomised controlled trial. Trials. 2017;18(1):284. doi: 10.1186/s13063-017-2003-7. PubMed PMID: 28629471.

29. Eissler S, Sanou A, Heckert J, Myers E, Nignan S, Thio E, et al. Gender dynamics, women’s empowerment, and diets: Qualitative findings from an impact evaluation of a nutrition-sensitive poultry value chain intervention in Burkina Faso. Washington, DC: International Food Policy Research Institute, 2020.

30. Gelli A, Headey D, Ngure F, Becquey E, Ganaba R, Huybregts L, et al. Assessing the health and nutrition risks of smallholder poultry production in Burkina Faso: Insights from formative research. Washington, DC: 2017.

31. SELEVER. LIVRET DE MESSAGES CLÉS SUR LA NUTRITION ET L’HYGIÈNE DESTINÉ AUX VULGARISATEURS VOLONTAIRES VILLAGEOIS. 2017.

32. SELEVER. Boites a images pour la promotion de l’équité genre dans la chaine de valeur volaille locale. 2017.

33. Leight J, Awonon J, Pedehombga A, Ganaba R, Martinez EM, Heckert J, et al. The impact of an integrated value chain intervention on household poultry production in Burkina Faso: Evidence from a randomized controlled trial. Washington, DC: International Food Policy Research Institute (IFPRI), 2020.

34. Becquey E, Diop L, Awonon J, Diatta A, D. , Ganaba R, Pedehombga A, et al. A Poultry Value Chain Intervention Promoting Diversified Diets Has Limited Impact on Maternal and Child Diet Adequacy during the Lean Season in a Cluster Randomized Controlled Trial. Journal of Nutrition. 2022;152(5):1336-46. doi: 10.1093/jn/nxac034. . PubMed Central PMCID: PMC9071289.

35. Gelli A, Collishaw A, Awonon J, Becquey E, Diatta A, Diop L, et al. Effects of an integrated poultry value chain, nutrition, gender and WASH intervention (SELEVER) on hygiene and child morbidity and anthropometry in Burkina Faso: A secondary outcome analysis of a cluster randomised trial. Matern Child Nutr. 2023;19(4):e13528. Epub 20230527. doi: 10.1111/mcn.13528. PubMed PMID: 37244872; PubMed Central PMCID: PMC10483954.

36. Berti PR, Heber Araujo C. Raising chickens for increased egg consumption in a rural highland Bolivian population. Food security. 2017;9(6):1329-41. doi: <https://dx.doi.org/10.1007/s12571-017-0728-5>.

37. Olney DK, Aminuzzaman T, Lora LI, Marie TR, Victoria Q. Assessing Impact and Impact Pathways of a Homestead Food Production Program on Household and Child Nutrition in Cambodia. Food and Nutrition Bulletin. 2009;30(4):355-69. doi: <http://dx.doi.org/10.1177/156482650903000407>. PubMed PMID: IND606218788 Pagination: p. 355-369. Identifiers: Children.

38. Olney DK, Vicheka S, Kro M, Chakriya C, Kroeun H, Hoing LS, et al. Using program impact pathways to understand and improve program delivery, utilization, and potential for impact of Helen Keller International's homestead food production program in Cambodia. Food Nutr Bull. 2013;34(2):169-84. doi: 10.1177/156482651303400206. PubMed PMID: 23964390.

39. Reinbott A, Jordan I. Determinants of Child Malnutrition and Infant and Young Child Feeding Approaches in Cambodia. World review of nutrition and dietetics. 2016;115:61-7. doi: 10.1159/000444609.

40. Reinbott A, Schelling A, Kuchenbecker J, Jeremias T, Russell I, Kevanna O, et al. Nutrition education linked to agricultural interventions improved child dietary diversity in rural Cambodia. British Journal of Nutrition. 2016;116(8):1457-68. doi: <http://dx.doi.org/10.1017/S0007114516003433>. PubMed PMID: IND606548855 Pagination: p. 1457-1468. Identifiers: Community-based nutrition.

41. Reinbott A, Schelling A, Jordan I, Krawinkel MB. Effectiveness of a nutrition education linked to agricultural interventions to improve infant and young child feeding practices, perceptions, and nutritional status in Cambodia. Maternal and Child Nutrition. 2018;14. doi: 10.1111/mcn.12587.

42. Michaux KD, Hou K, Karakochuk CD, Whitfield KC, Ly S, Verbowski V, et al. Effect of enhanced homestead food production on anaemia among Cambodian women and children: A cluster randomized controlled trial. Maternal and Child Nutrition. 2019;15(S3). doi: 10.1111/mcn.12757.

43. Verbowski V, Talukder Z, Hou K, Sok Hoing L, Michaux K, Anderson V, et al. Effect of enhanced homestead food production and aquaculture on dietary intakes of women and children in rural Cambodia: A cluster randomized controlled trial. Matern Child Nutr. 2018;14(3):e12581. Epub 2018/01/10. doi: 10.1111/mcn.12581. PubMed PMID: 29314705; PubMed Central PMCID: PMC6866186.

44. Dragojlovic N, Michaux KD, Moumin NA, Li KH, Talukder Z, Hou K, et al. Economic evaluation of an enhanced homestead food production intervention for undernutrition in women and children in rural Cambodia. Global Food Security-Agriculture Policy Economics and Environment. 2020;24. doi: 10.1016/j.gfs.2019.100335. PubMed PMID: WOS:000529332000010.

45. Moumin NA, Kroeun H, Michaux KD, Stormer A, Mundy G, Porter K, et al. Impact of an enhanced homestead food production program on household food production and dietary intake of women aged 15-49 years and children aged 6-59 months: a pragmatic delayed cluster randomized control trial protocol. International Journal of Clinical Trials. 2017;4(4):157-65. doi: <http://dx.doi.org/10.18203/2349-3259.ijct20174863>.

46. Ji G, Qi R, Wang H, Feng C, Leng J. A planting and eating soybean project for people living with HIV/AIDS in rural Anhui - A pilot study in China. AIDS Care - Psychological and Socio-Medical Aspects of AIDS/HIV. 2010;22(1):126-32. doi: 10.1080/09540120903012536.

47. Vasquez TM, Del Castillo S, Galvez DC, Rodriguez LE. Breeding Differently: Participatory Selection and Scaling Up Innovations in Colombia. Potato Research. 2017;60(3-4):361-81. doi: 10.1007/s11540-018-9389-9. PubMed PMID: WOS:000443393800015.

48. Doocy S, Amundson-Mansen K, Colantouni E, Emerson J, Menakuntuala J, Strong J. Evaluating interventions to improve child nutrition in Eastern Democratic Republic of Congo. Public Health Nutrition. 2019;22(1):3-14. doi: <http://dx.doi.org/10.1017/S1368980018002859>. PubMed PMID: IND606515432 Pagination: p. 3-14. Identifiers: Dietary diversity.

49. Doocy S, Amundson K. Jenga Jamaa II Operations Research. 2016.

50. JENGA II Nutrition Counseling Training. 2013.

51. Care Group Criteria Small Group Questions. n.d.

52. Klemm RDW, Burns J, Amundson K. Formative Research to Examine Perceptions and Behaviors about Maternal, Infant and Young Child Feeding - JENGA JAMAA II, Democratic Republic of Congo. 2012.

53. Formative Research Results - Jenga Jamaa II Presentation. 2012.

54. Klemm R, Burns J, Amundson K. Formative Research to Examine Perceptions and Behaviors about Maternal, Infant, and Young Child Feedng - JENGA JAMAA II - Preliminary Report of Findings. n.d.

55. JENGA II Training: Health, Nutrition, Care Groups and Behavior Change Strategy. 2011.

56. Ayele Z, Peacock C. Improving access to and consumption of animal source foods in rural households: the experiences of a women-focused goat development program in the highlands of Ethiopia. J Nutr. 2003;133(11 Suppl 2):3981s-6s. doi: 10.1093/jn/133.11.3981S. PubMed PMID: 14672299.

57. Kim SS, Nguyen PH, Yohannes Y, Abebe Y, Tharaney M, Drummond E, et al. Behavior Change Interventions Delivered through Interpersonal Communication, Agricultural Activities, Community Mobilization, and Mass Media Increase Complementary Feeding Practices and Reduce Child Stunting in Ethiopia. J Nutr. 2019;149(8):1470-81. doi: 10.1093/jn/nxz087. PubMed PMID: 31165869; PubMed Central PMCID: PMC6686053.

58. Kim SS, Nguyen PH, Tran LM, Alayon S, Menon P, Frongillo EA. Different Combinations of Behavior Change Interventions and Frequencies of Interpersonal Contacts Are Associated with Infant and Young Child Feeding Practices in Bangladesh, Ethiopia, and Vietnam. Curr Dev Nutr. 2020;4(2):nzz140. Epub 20191209. doi: 10.1093/cdn/nzz140. PubMed PMID: 31976385; PubMed Central PMCID: PMC6964730.

59. Moss C, Bekele TH, Salasibew MM, Sturgess J, Ayana G, Kuche D, et al. Sustainable Undernutrition Reduction in Ethiopia (SURE) evaluation study: a protocol to evaluate impact, process and context of a large-scale integrated health and agriculture programme to improve complementary feeding in Ethiopia. BMJ Open. 2018;8(7):e022028. doi: 10.1136/bmjopen-2018-022028. PubMed PMID: 30030320.

60. Salasibew MM, Moss C, Ayana G, Kuche D, Eshetu S, Dangour AD. The fidelity and dose of message delivery on infant and young child feeding practice and nutrition sensitive agriculture in Ethiopia: a qualitative study from the Sustainable Undernutrition Reduction in Ethiopia (SURE) programme. Journal of health, population, and nutrition. 2019;38(1):29. doi: 10.1186/s41043-019-0187-z.

61. Worku T, Gonete KA, Muhammad EA, Atnafu A. Sustainable under nutrition reduction program and dietary diversity among children’s aged 6–23 months, Northwest Ethiopia: Comparative cross-sectional study. International Journal for Equity in Health. 2020;19(1):14. doi: 10.1186/s12939-019-1120-1.

62. Passarelli S, Ambikapathi R, Gunaratna NS, Madzorera I, Canavan CR, Noor AR, et al. A Chicken Production Intervention and Additional Nutrition Behavior Change Component Increased Child Growth in Ethiopia: A Cluster-Randomized Trial. J Nutr. 2020;150(10):2806-17. Epub 2020/07/12. doi: 10.1093/jn/nxaa181. PubMed PMID: 32652012; PubMed Central PMCID: PMC7549301.

63. Ambikapathi R, Passarelli S, Madzorera I, Canavan CR, Noor RA, Abdelmenan S, et al. Men's nutrition knowledge is important for women's and children's nutrition in Ethiopia. Matern Child Nutr. 2021;17(1):e13062. Epub 2020/08/06. doi: 10.1111/mcn.13062. PubMed PMID: 32755057; PubMed Central PMCID: PMC7729551.

64. Omer A, Hailu D, Whiting SJ. Egg Consumption of Children under Two Years of Age through a Child-Owned Poultry and Nutrition Intervention in Rural Ethiopia: A Community-Based Randomized Controlled Trial. Journal of Agricultural & Food Research. 2022;9(100354).

65. Omer A, Hailu D, Whiting SJ. Effect of a Child-Owned Poultry Intervention Providing Eggs on Nutrition Status and Motor Skills of Young Children in Southern Ethiopia: A Cluster Randomized and Controlled Community Trial. International Journal of Environmental Research and Public Health. 2022;19(15305).

66. Omer A, Hailu D, Whiting SJ. Child-Owned Poultry Intervention Effects on Hemoglobin, Anemia, Concurrent Anemia and Stunting, and Morbidity Status of Young Children in Southern Ethiopia: A Cluster Randomized Controlled Community Trial. Int J Environ Res Public Health. 2023;20(7). Epub 2023/04/14. doi: 10.3390/ijerph20075406. PubMed PMID: 37048019; PubMed Central PMCID: PMC10094074.

67. Glover-Amengor M, Agbemafle I, Hagan LL, Mboom FP, Gamor G, Larbi A, et al. Nutritional status of children 0-59 months in selected intervention communities in northern Ghana from the africa RISING project in 2012. Arch Public Health. 2016;74:12. doi: 10.1186/s13690-016-0124-1. PubMed PMID: 27047661.

68. Singoreli S, Haile, B., Kotu, B. Exploring the Agriculture-Nutrition Linkage in Northern Ghana. IFPRI Discussion Paper 16972017.

69. Saaka M, Mutaru, S. Integrating agricultural and nutritional Interventions for improved nutritional status of rural families in northern Ghana: Baseline survey report. 2013.

70. Saaka M, Osman SM, Hoeschle-Zeledon I. Relationship between agricultural biodiversity and dietary diversity of children aged 6-36 months in rural areas of Northern Ghana. Food Nutr Res. 2017;61(1):1391668. Epub 2017/11/21. doi: 10.1080/16546628.2017.1391668. PubMed PMID: 29151832; PubMed Central PMCID: PMC5678439.

71. Marquis G, Colecraft E, Aryeetey R, Lartey A, Clark S, Aboud F, et al. Nutrition links-building capacity for sustainable lives in Ghana. FASEB Journal. 2015;29(1). <http://www.embase.com/search/results?subaction=viewrecord&from=export&id=L71863945>.

72. Marquis GS, Colecraft EK, Kanlisi R, Aidam BA, Atuobi-Yeboah A, Pinto C, et al. An agriculture–nutrition intervention improved children's diet and growth in a randomized trial in Ghana. Maternal and Child Nutrition. 2018;14. doi: 10.1111/mcn.12677.

73. Palmer T, Darabian N. Farmers’ Club: A mobile agriculture service by Vodafone Ghana. GSMA, 2017.

74. Hidrobo M, Palloni G, Aker JC, Daniel GO, Ledlie N. Paying for Digital Information: Assessing Farmers’ Willingness to Pay for a Digital Agriculture and Nutrition Service in Ghana. IFRI Discussion Paper 19062020.

75. Guzmán-Abril A, Alajajian S, Rohloff P, Proaño GV, Brewer J, Jimenez EY. Academy of Nutrition and Dietetics Nutrition Research Network: A Home Garden Intervention Improves Child Length-for-Age Z-Score and Household-Level Crop Count and Nutritional Functional Diversity in Rural Guatemala. J Acad Nutr Diet. 2022;122(3):640-9.e12. Epub 2021/05/23. doi: 10.1016/j.jand.2021.04.002. PubMed PMID: 34020932.

76. Birdi TJ, Joshi S, Kotian S, Shah S. Possible causes of malnutrition in Melghat, a tribal region of Maharashtra, India. Glob J Health Sci. 2014;6(5):164-73. Epub 2014/08/30. doi: 10.5539/gjhs.v6n5p164. PubMed PMID: 25168997; PubMed Central PMCID: PMC4825484.

77. Birdi TJ, Shah SU. Implementing Perennial Kitchen Garden Model to Improve Diet Diversity in Melghat, India. Glob J Health Sci. 2015;8(4):10-21. doi: 10.5539/gjhs.v8n4p10. PubMed PMID: 26573040.

78. Chinai R, Birdi T. Policy Brief: Perennial Kitchen Gardens - A solution for Melghat malnutrition. Maharashtra, India: The Foundations for Medical Research, n.d.

79. Unknown. How Does Your Garden Grow: Project Video. n.d.

80. Haghparast-Bidgoli H, Skordis J, Harris-Fry H, Krishnan S, O'Hearn M, Kumar A, et al. Protocol for the cost-consequence and equity impact analyses of a cluster randomised controlled trial comparing three variants of a nutrition-sensitive agricultural extension intervention to improve maternal and child dietary diversity and nutritional status in rural Odisha, India (UPAVAN trial). Trials. 2019;20(1). doi: 10.1186/s13063-019-3388-2.

81. Harris-Fry H, Krishnan S, Beaumont E, Prost A, Gouda S, Mohanty S, et al. Agricultural and empowerment pathways from land ownership to women's nutrition in India. Maternal and Child Nutrition. 2020. doi: 10.1111/mcn.12995. PubMed PMID: WOS:000520728700001.

82. Kadiyala S, Morgan EH, Cyriac S, Margolies A, Roopnaraine T. Adapting agriculture platforms for nutrition: A case study of a participatory, video-based agricultural extension platform in India. PLoS ONE. 2016;11(10). doi: 10.1371/journal.pone.0164002.

83. Kadiyala S, Prost A, Harris-Fry H, O'Hearn M, Pradhan R, Pradhan S, et al. Upscaling Participatory Action and Videos for Agriculture and Nutrition (UPAVAN) trial comparing three variants of a nutrition-sensitive agricultural extension intervention to improve maternal and child nutritional outcomes in rural Odisha, India: Study protocol for a cluster randomised controlled trial. Trials. 2018;19(1). doi: 10.1186/s13063-018-2521-y.

84. Harris-Fry H, O'Hearn M, Pradhan R, Krishnan S, Nair N, Rath S, et al. How to design a complex behaviour change intervention: experiences from a nutrition-sensitive agriculture trial in rural India. BMJ Glob Health. 2020;5(6). Epub 2020/06/10. doi: 10.1136/bmjgh-2020-002384. PubMed PMID: 32513863; PubMed Central PMCID: PMC7282327.

85. Aakesson A, Cunningham S, Danton H, Granger K, Harris-Fry H, Hogan S, et al. UPAVAN Formative Research Report. Arlington, VA: Strengthening Partnerships, Results, and Innovations in Nutrition Globally (SPRING) project, 2017.

86. Kadiyala S, Harris-Fry H, Pradhan R, Satyanarayan M, Pradhan S, Rath S, et al. Upscaling Participatory Action and Videos for Agriculture and Nutrition (UPAVAN) trial in rural Odisha, India: An overview of results and lessons learnt (presentation). 2020.

87. Kadiyala S, Harris-Fry H, Pradhan R, Mohanty S, Padhan S, Rath S, et al. Effect of nutrition-sensitive agriculture interventions with participatory videos and women's group meetings on maternal and child nutritional outcomes in rural Odisha, India (UPAVAN trial): a four-arm, observer-blind, cluster-randomised controlled trial. Lancet Planet Health. 2021;5(5):e263-e76. Epub 2021/04/04. doi: 10.1016/s2542-5196(21)00001-2. PubMed PMID: 33811818; PubMed Central PMCID: PMC8099729.

88. Haghparast-Bidgoli H, Harris-Fry H, Kumar A, Pradhan R, Mishra NK, Padhan S, et al. Economic Evaluation of Nutrition-Sensitive Agricultural Interventions to Increase Maternal and Child Dietary Diversity and Nutritional Status in Rural Odisha, India. J Nutr. 2022;152(10):2255-68. Epub 2022/06/11. doi: 10.1093/jn/nxac132. PubMed PMID: 35687367; PubMed Central PMCID: PMC9535442.

89. Kalavathi S, Krishnakumar VP, Thomas RJ, Thomas GV, George ML. Improving food and nutritional security of small and marginal coconut growers through diversification of crops and enterprises. Journal of Agriculture and Rural Development in the Tropics and Subtropics. 2010;111(2):101-9. <Go to ISI>://WOS:000297185600004. PubMed PMID: WOS:000297185600004.

90. Murty PVVS, Rao MV, Bamji MS. Impact of Enriching the Diet of Women and Children Through Health and Nutrition Education, Introduction of Homestead Gardens and Backyard Poultry in Rural India. Agric Res. 2016;5:210-7. doi: <https://doi.org/10.1007/s40003-016-0206-x>.

91. Dangoria Charitable Trust Annual Report 2014-2015. Hyderbad, India: Dangoria Charitable Trust, 2015.

92. Bhaskar AVV, Nithya DJ, Raju S, Bhavani RV. Establishing integrated agriculture-nutrition programmes to diversify household food and diets in rural India. Food security. 2017;9(5):981-99. doi: 10.1007/s12571-017-0721-z. PubMed PMID: WOS:000414999200007.

93. Narayanan S, Rao N. Adult learning for nutrition security: Challenging dominant values through participatory action research in Eastern India. Studies in Education of Adults. 2019;51(2):213-31. doi: 10.1080/02660830.2019.1573782.

94. Narayanan S, Nayak TR, Swain SK, Tosh R. Towards Nutrition Security: Community Hunger Fighters - Toolkit for Implementation. 2015.

95. Narayanan R, Nayak TR. Towards Nutrition Security: Community Hunger Figthers Programme - Residential Training Programme on Food and Nutrition Security. M. S. Swaminathan Research Foundation, 2015.

96. Narayanan R, Nithya DJ, Panda AK, Wagh RD. Community Hunger Fighters: An Adult Nutrition Literacy Programme (Experiences in integrating nutrition literacy as part of the Farming system for Nutrition Study under the research programme on Leveraging Agriculture for Nutrition in South Asia). M S Swaminathan Research Foundation, 2018.

97. Rajeesh S, Nithya DJ, Raju S, Bhavani RV. India Impact Brief: Nutrition awareness under Farming System for Nutrition study. n.d.

98. Boedecker J, Odhiambo Odour F, Lachat C, Van Damme P, Kennedy G, Termote C. Participatory farm diversification and nutrition education increase dietary diversity in Western Kenya. Matern Child Nutr. 2019;15(3):e12803. doi: 10.1111/mcn.12803. PubMed PMID: 30827036.

99. International B. Methodology of door-to-door nutrition education in Vihiga County, Western Kenya. n.d.

100. Boedecker J, Termote C, Kennedy G. Participatory development of agricultural interventions to improve nutrition at community level: A Manual based on participatory intervention development in Western Kenya. n.d.

101. International B. Methodology for the cooking sessions. n.d.

102. Cole DC, Levin C, Loechl C, Thiele G, Grant F, Girard AW, et al. Planning an integrated agriculture and health program and designing its evaluation: Experience from Western Kenya. Evaluation and program planning. 2016;56:11-22. doi: 10.1016/j.evalprogplan.2016.03.001.

103. Girard AW, Grant F, Watkinson M, Okuku HS, Wanjala R, Cole D, et al. Promotion of orange-fleshed sweet potato increased vitamin A intakes and reduced the odds of low retinol-binding protein among postpartum Kenyan women. Journal of Nutrition. 2017;147(5):955-63. doi: 10.3945/jn.116.236406.

104. SASHA Proof-of-Concept Project Kenya Agriculture-Health Linkages - Organizational Strategy to Guide Project Implementation. n.d.

105. Machira Y. Technical Report - SASHA Kenya Agriculture-Health Proof-of-Concept Project Operations Research for the Pilot Implementation. 2011.

106. Machira Y. Exploring the Acceptability and Feasibility of Integrating Vitamin A Rich Sweetpotato into AnteNatal Care Services in Western Kenya: Results from Operations Research Conducted during the Implementation Phase of the Mama SASHA Project. International Potato Center (CIP), 2018.

107. Ouedraogo H. Mama SASHA Baseline Survey Report. Nairobi, Kenya: PATH & International Potato Center (CIP), 2011.

108. Webb Girard A, Brouwer A, Faerber E, Grant FK, Low J. Orange-fleshed sweetpotato: Strategies and lessons learned for achieving food security and health at scale in Sub-Saharan Africa. Open Agriculture. 2021;6(1):511-36. doi: doi:10.1515/opag-2021-0034.

109. Okello JJ, Kwikiriza N, Muoki P, Wambaya J, Heck S. Effect of Intensive Agriculture-Nutrition Education and Extension Program Adoption and Diffusion of Biofortified Crops. Journal of Agricultural & Food Information. 2019;20(3):254-76. doi: 10.1080/10496505.2018.1515632. PubMed PMID: WOS:000482360500006.

110. Wegmüller R, Musau K, Vergari L, Custer E, Anyango H, Donkor WES, et al. Effectiveness of an integrated agriculture, nutrition-specific, and nutrition-sensitive program on child growth in Western Kenya: a cluster-randomized controlled trial. Am J Clin Nutr. 2022;116(2):446-59. Epub 2022/04/15. doi: 10.1093/ajcn/nqac098. PubMed PMID: 35421217; PubMed Central PMCID: PMC9348977.

111. Petry N, Wegmuller R, Rohner F. Clinical Study Protocol: Effectiveness of an integrated programme to reduce maternal and child malnutrition in Kenya. Available at: <https://osfio/y364a> 2020.

112. Ojwang’ SO, Okello JJ, Otieno DJ, Nyikal RA, Muoki PN. Using preschoolers to improve caregivers' knowledge, attitude, and practices relating to biofortified crops: Evidence from a randomized nutrition education trial in Kenya. Food Science & Nutrition. 2022;10:3627–37. doi: <https://doi.org/10.1002/fsn3.2960>.

113. Ojwang SO, Otieno DJ, Okello JJ, Muoki P, Nyikal RA. Do Nutrition Education Approaches With Preschoolers and Their Caregivers Influence Retention of Biofortified Orange-Fleshed Sweet Potato on Farms? Evidence From Homa Bay County, Kenya. Food Nutr Bull. 2021;42(3):347-60. Epub 2021/07/07. doi: 10.1177/03795721211025445. PubMed PMID: 34225479.

114. Gelli A, Margolies A, Santacroce M, Roschnik N, Twalibu A, Katundu M, et al. Using a Community-Based Early Childhood Development Center as a Platform to Promote Production and Consumption Diversity Increases Children's Dietary Intake and Reduces Stunting in Malawi: A Cluster-Randomized Trial. J Nutr. 2018;148(10):1587-97. doi: 10.1093/jn/nxy148. PubMed PMID: 30204916.

115. Gelli A, Nguyen PH, Santacroce M, Twalibu A, Margolies A, Katundu M. A Community-Based Early Childhood Development Center Platform Promoting Diversified Diets and Food Production Increases the Mean Probability of Adequacy of Intake of Preschoolers in Malawi: A Cluster Randomized Trial. The Journal of nutrition. 2019. doi: 10.1093/jn/nxz245.

116. Margolies A, Gelli A, Daryanani R, Twalibu A, Levin C. When Communities Pull Their Weight: The Economic Costs of an Integrated Agriculture and Nutrition Home-Grown Preschool Meal Intervention in Malawi. Food Nutr Bull. 2021;42(1):3-22. doi: 10.1177/0379572120986693. PubMed PMID: 33878905; PubMed Central PMCID: PMC8129463.

117. Gelli A, Kemp CG, Margolies A, Twalibu A, Katundu M, Levin C. Economic evaluation of an early childhood development center– based agriculture and nutrition intervention in Malawi. . Food Security. 2022;14(1):67–80.

118. Bezner Kerr R, Berti PR, Shumba L. Effects of a participatory agriculture and nutrition education project on child growth in northern Malawi. Public Health Nutr. 2011;14(8):1466-72. doi: 10.1017/s1368980010002545. PubMed PMID: 21059284.

119. Bezner Kerr R, Shumba L, Msachi R, Snapp S, Chirwa M. Participatory research on legume diversification with Malawian smallholder farmers for improved human nutrition and soil fertility [electronic resource]. Experimental agriculture. 2007;43(4):437-53. doi: <http://dx.doi.org/10.1017/S0014479707005339>. PubMed PMID: IND43978036 Pagination: p. 437-453. Publicaton Type: Journal Article Language(s): English Bibliography Statement: Includes references Category Codes: Nutrition and Health Education.

120. Satzinger F, Bezner Kerr R, Shumba L. Intergenerational participatory discussion groups foster knowledge exchange to improve child nutrition and food security in northern Malawi. Ecol Food Nutr. 2009;48(5):369-82. doi: 10.1080/03670240903170483. PubMed PMID: 21883064.

121. Kerr RB, Chilanga E, Nyantakyi-Frimpong H, Luginaah I, Lupafya E. Integrated agriculture programs to address malnutrition in northern Malawi. BMC Public Health. 2016;16(1):1197. doi: 10.1186/s12889-016-3840-0. PubMed PMID: 27894303.

122. Kangmennaang J, Kerr RB, Lupafya E, Dakishoni L, Katundu M, Luginaah I. Impact of a participatory agroecological development project on household wealth and food security in Malawi. Food security. 2017;9(3):561-76. doi: 10.1007/s12571-017-0669-z. PubMed PMID: WOS:000403459800012.

123. Kerr RB, Kangmennaang J, Dakishoni L, Nyantakyi-Frimpong H, Lupafya E, Shumba L, et al. Participatory agroecological research on climate change adaptation improves smallholder farmer household food security and dietary diversity in Malawi. Agriculture Ecosystems & Environment. 2019;279:109-21. doi: 10.1016/j.agee.2019.04.004. PubMed PMID: WOS:000482173600013.

124. Muehlhoff E, Wijesinha-Bettoni R, Westaway E, Jeremias T, Nordin S, Garz J. Linking agriculture and nutrition education to improve infant and young child feeding: Lessons for future programmes. Maternal and Child Nutrition. 2017;13. doi: 10.1111/mcn.12411. PubMed PMID: WOS:000412989600002.

125. Kuchenbecker J, Reinbott A, Mtimuni B, Krawinkel MB, Jordan I. Nutrition education improves dietary diversity of children 6-23 months at community-level: Results from a cluster randomized controlled trial in Malawi. PLoS ONE. 2017;12(4):e0175216. doi: 10.1371/journal.pone.0175216. PubMed PMID: 28426678.

126. De Brauw A, Eozenou P, Moursi M. Programme Participation Intensity and Children's Nutritional Status: Evidence from a Randomised Control Trial in Mozambique. Journal of Development Studies. 2015;51(8):996-1015. doi: 10.1080/00220388.2015.1018907. PubMed PMID: WOS:000360613300004.

127. Hotz C, Loechl C, de Brauw A, Eozenou P, Gilligan D, Moursi M, et al. A large-scale intervention to introduce orange sweet potato in rural Mozambique increases vitamin A intakes among children and women. British Journal of Nutrition. 2012;108(1):163-76. doi: <https://dx.doi.org/10.1017/S0007114511005174>. PubMed PMID: IND605162159 Pagination: p. 163-176. Identifiers: Orange sweet potato.

128. Low JW, Arimond M, Osman N, Cunguara B, Zano F, Tschirley D. Ensuring the supply of and creating demand for a biofortified crop with a visible trait: Lessons learned from the introduction of orange-fleshed sweet potato in drought-prone areas of Mozambique. Food and Nutrition Bulletin. 2007;28(2 SUPPL.):S258-S70. <http://www.embase.com/search/results?subaction=viewrecord&from=export&id=L46916855>.

129. Broaddus-Shea ET, Shrestha BT, Rana PP, Winch PJ, Underwood CR. Navigating structural barriers to the implementation of agriculture-nutrition programs in Nepal. Food security. 2020;12(3679-690). doi: 10.1007/s12571-020-01031-0. PubMed PMID: WOS:000527450200001.

130. Dulal B, Gary M, Kenda C, Pooja Pandey R, Rojee S. Homestead Food Production and Maternal and Child Dietary Diversity in Nepal: Variations in Association by Season and Agroecological Zone. Food and Nutrition Bulletin. 2017;38(3):338-53. doi: <http://dx.doi.org/10.1177/0379572117703264>. PubMed PMID: IND606218401 Pagination: p. 338-353. Identifiers: dietary diversity.

131. Malapit HJL, Kadiyala S, Quisumbing AR, Cunningham K, Tyagi P. Women’s empowerment in agriculture, production diversity, and nutrition: Evidence from Nepal. IFPRI Discussion Paper 13132013.

132. HKI. Suahara II: Social and Behavior Change Strategy. n.d.

133. HKI. SUAAHARA II: Social and Behavior Change (SBCC) Technical Brief. 2018.

134. HKI. Social and Behavior Change Implementation Brief: Suaahara II. n.d.

135. HKI. Suaahara II Egg Promotion Campaign strategy Paper and Implementation Guide. 2020.

136. HKI. Formative Research: Exploring factors affecting key Suaahara II behaviors and ways to address them. 2019.

137. USAID. Mid-Term Performance Evaluation of USAID/Nepal’s SUAAHARA II Integrated Nutrition Program United States Agency for International Development (USAID), 2019.

138. Jones KM, Kenneth HB, Lindsay HA, Parvati S, Sheila ES. Nutrition Knowledge and Practices, and Consumption of Vitamin A–Rich Plants by Rural Nepali Participants and Nonparticipants in a Kitchen-Garden Program. Food and Nutrition Bulletin. 2005;26(2):198-208. doi: <http://dx.doi.org/10.1177/156482650502600204> <https://handle.nal.usda.gov/10113/44449>. PubMed PMID: IND44424078 Pagination: p. 198-208. Identifiers: Adult.

139. Miller LC, Neupane S, Joshi N, Lohani M, Rogers BL, Neupane S, et al. Multisectoral community development in Nepal has greater effects on child growth and diet than nutrition education alone. Public Health Nutr. 2020;23(1):146-61. Epub 20190923. doi: 10.1017/s136898001900260x. PubMed PMID: 31544735; PubMed Central PMCID: PMC10200641.

140. Osei AK, Pandey P, Spiro D, Adhikari D, Haselow N, De Morais C, et al. Adding multiple micronutrient powders to a homestead food production programme yields marginally significant benefit on anaemia reduction among young children in Nepal. Maternal and Child Nutrition. 2015;11:188-202. doi: 10.1111/mcn.12173.

141. Osei A, Alissa P, Dale D, David S, Jennifer N, Nancy H, et al. Combining Home Garden, Poultry, and Nutrition Education Program Targeted to Families With Young Children Improved Anemia Among Children and Anemia and Underweight Among Nonpregnant Women in Nepal. Food and Nutrition Bulletin. 2017;38(1):49-64. doi: <http://dx.doi.org/10.1177/0379572116676427>. PubMed PMID: IND606218377 Pagination: p. 49-64. Identifiers: growth.

142. Kjeldsberg C, Shrestha N, Patel M, Davis D, Cunningham K. Nutrition-sensitive agricultural interventions and gender dynamics: A qualitative study in Nepal. Maternal and Child Nutrition. 2018;14. doi: 10.1111/mcn.12587.

143. Locks LM, Pandey PR, Osei AK, Spiro DS, Adhikari DP, Haselow NJ, et al. Using formative research to design a context-specific behaviour change strategy to improve infant and young child feeding practices and nutrition in Nepal. Matern Child Nutr. 2015;11(4):882-96. Epub 2013/04/06. doi: 10.1111/mcn.12032. PubMed PMID: 23557321; PubMed Central PMCID: PMC6860308.

144. Flax V, Ouma E, Poole J, Izerimana L, King G. Enhancing milk quality and consumption for improved income and nutrition in Rwanda: Nutrition baseline survey report. . Gainesville, FL, USA: Feed the Future Innovation Lab for Livestock Systems, 2019.

145. Flax VL, Ouma E, Izerimana L, Schreiner MA, Brower AO, Niyonzima E, et al. Animal Source Food Social and Behavior Change Communication Intervention Among Girinka Livestock Transfer Beneficiaries in Rwanda: A Cluster Randomized Evaluation. Glob Health Sci Pract. 2021;9(3):640-53. Epub 20210930. doi: 10.9745/GHSP-D-21-00082. PubMed PMID: 34593587; PubMed Central PMCID: PMC8514034.

146. Flax VL, Ouma EA, Schreiner MA, Ufitinema A, Niyonzima E, Colverson KE, et al. Engaging fathers to support child nutrition increases frequency of children's animal source food consumption in Rwanda. PLoS One. 2023;18(4):e0283813. Epub 2023/04/08. doi: 10.1371/journal.pone.0283813. PubMed PMID: 37027367; PubMed Central PMCID: PMC10081762.

147. Nordhagen S, Nielsen J, van Mourik T, Smith E, Klemm R. Fostering CHANGE: Lessons from implementing a multi-country, multi-sector nutrition-sensitive agriculture project. Eval Program Plann. 2019;77:101695. Epub 20190731. doi: 10.1016/j.evalprogplan.2019.101695. PubMed PMID: 31400655.

148. Nordhagen S, Thiam K, Sow S. The sustainability of a nutrition-sensitive agriculture intervention: a case study from urban Senegal. Food Security. 2019;11(5):1121-34. doi: 10.1007/s12571-019-00948-5.

149. Nordhagen S, Klemm R. Implementing small-scale poultry-for-nutrition projects: Successes and lessons learned. Maternal and Child Nutrition. 2018;14. doi: 10.1111/mcn.12676.

150. Faber M, Laurie S, Thompson B, Amoroso L. A Home Gardening Approach Developed in South Africa to Address Vitamin A Deficiency2011. 163-82 p.

151. Faber M, Dhansay MA, Benad©♭ AJS, Phungula MAS, Venter SL. Home gardens focusing on the production of yellow and dark-green leafy vegetables increase the serum retinol concentrations of 2–5-y-old children in South Africa. American Journal of Clinical Nutrition. 2002;76(5):1048-54. doi: <http://www.ajcn.org/content/76/5.toc>.

152. Laurie SM, Faber M. Integrated community-based growth monitoring and vegetable gardens focusing on crops rich in Îø-carotene: Project evaluation in a rural community in the Eastern Cape, South Africa [electronic resource]. Journal of the Science of Food and Agriculture. 2008;88(12):2093-101. doi: <http://dx.doi.org/10.1002/jsfa.3319>. PubMed PMID: IND44094131 Pagination: p. 2093-2101. Publicaton Type: Journal Article Language(s): English Bibliography Statement: Includes references Category Codes: Nutrition and Health Education.

153. Bonuedi I, Kornher L, Gerber N. Making Cash Crop Value Chains Nutrition-Sensitive: Evidence from a Quasi-Experiment in Rural Sierra Leone. ZEF Discussion Papers on Development Policy. Bonn, Germany: Center for Development Research; 2020. p. 54.

154. Blakstad MM, Bellows AL, Canavan CR, Fawzi WW, Kinabo J, Kruk ME, et al. Neighbour home gardening predicts dietary diversity among rural Tanzanian women. Public Health Nutrition. 2019;22(9):1646-53. doi: <http://dx.doi.org/10.1017/S1368980018003798>. PubMed PMID: IND606523752 Pagination: p. 1646-1653. Identifiers: Agriculture.

155. Mosha D, Canavan CR, Bellows AL, Blakstad MM, Noor RA, Masanja H, et al. The impact of integrated nutrition-sensitive interventions on nutrition and health of children and women in rural Tanzania: study protocol for a cluster-randomized controlled trial. BMC Nutr. 2018;4:29. Epub 2018/09/06. doi: 10.1186/s40795-018-0238-7. PubMed PMID: 32153890; PubMed Central PMCID: PMC7050733.

156. Blakstad MM, Mosha D, Bellows AL, Canavan CR, Chen JT, Mlalama K, et al. Home gardening improves dietary diversity, a cluster-randomized controlled trial among Tanzanian women. Matern Child Nutr. 2021;17(2):e13096. Epub 2020/11/27. doi: 10.1111/mcn.13096. PubMed PMID: 33241924; PubMed Central PMCID: PMC7988851.

157. Blakstad MM, Mosha D, Bliznashka L, Bellows AL, Canavan CR, Yussuf MH, et al. Are home gardening programs a sustainable way to improve nutrition? Lessons from a cluster-randomized controlled trial in Rufiji, Tanzania. Food Policy. 2022;109. Epub 20220321. doi: 10.1016/j.foodpol.2022.102248. PubMed PMID: 35431402; PubMed Central PMCID: PMC9012451.

158. Kidala D, Greiner T, Gebre-Medhin M. Five-year follow-up of a food-based vitamin A intervention in Tanzania. Public Health Nutr. 2000;3(4):425-31. doi: 10.1017/s1368980000000495. PubMed PMID: 11135797.

159. Santoso MV, Bezner Kerr RN, Kassim N, Martin H, Mtinda E, Njau P, et al. A Nutrition-Sensitive Agroecology Intervention in Rural Tanzania Increases Children's Dietary Diversity and Household Food Security But Does Not Change Child Anthropometry: Results from a Cluster-Randomized Trial. J Nutr. 2021;151(7):2010-21. Epub 2021/05/12. doi: 10.1093/jn/nxab052. PubMed PMID: 33973009; PubMed Central PMCID: PMC8245885.

160. Bezner Kerr R, Young SL, Young C, Santoso MV, Malagasi M, Entz M, et al. Farming for change: Developing a participatory curriculum on agroecology, nutrition, climate change and social equity in Malawi and Tanzania. Agriculture and human values. 2019;36:549-66. doi: <https://doi.org/10.1007/s10460-018-09906-x>.

161. Ochieng J, Afari-Sefa V, Karanja D, Kessy R, Rajendran S, Samali S. How promoting consumption of traditional African vegetables affects household nutrition security in Tanzania. Renewable Agriculture and Food Systems. 2018;33(2):105-15. doi: 10.1017/s1742170516000508. PubMed PMID: WOS:000426673400001.

162. Roesler A. Development and sustainability of interventions to improve child nutrition: a case-study of ethnic minority communities in northern Thailand: University of Adelaide; 2018.

163. Roesler AL, Smithers LG, Wangpakapattanawong P, Moore V. Stunting, dietary diversity and household food insecurity among children under 5 years in ethnic communities of northern Thailand. J Public Health (Oxf). 2019;41(4):772-80. Epub 2018/11/14. doi: 10.1093/pubmed/fdy201. PubMed PMID: 30423144.

164. Roesler A, Smithers LG, Winichagoon P, Wangpakapattanawong P, Moore V. Local perspectives and context in relation to feeding practices of children under 2 years in the mountain villages of northern Thailand. Public Health Nutr. 2018;21(16):2989-97. Epub 2018/08/18. doi: 10.1017/S1368980018001957. PubMed PMID: 30115134.

165. Berti PR, Desrochers RE, Van HP, Van AL, Ngo TD, The KH, et al. The process of developing a nutrition-sensitive agriculture intervention: a multi-site experience. Food security. 2016;8(6):1053-68. doi: 10.1007/s12571-016-0625-3. PubMed PMID: WOS:000389897200004.

166. Major A. Nutrition-sensitive agriculture programming: addressing demand- and supply-side factors in Timor-Leste. Crawford Fund Annual Conference 2018: AgEcon; 2018. p. 94-9.

167. TOMAK. TOMAK To’os ba Moris Di’ak Farming for Prosperity: Program Guiding Strategy. 2016.

168. Meyanathan S. TOMAK Social and Behavior Change Strategy. 2019.

169. TOMAK. TOMAK Component 1 Midline Evaluation Full Report. 2021.

170. Hotz C, Loechl C, Lubowa A, Tumwine JK, Masawi GN, Baingana R, et al. Introduction of β-Carotene-Rich orange sweet potato in rural Uganda resulted in increased vitamin a intakes among children and women and improved vitamin a status among children. Journal of Nutrition. 2012;142(10):1871-80. doi: 10.3945/jn.111.151829.

171. Shaw K, Ngoc PK, Lan H, Mundy G, Talukder Z. How non-targeted households benefited from implementing a homestead food production program in Son La, Vietnam. Diversity and change in food wellbeing. 2018. p. 267-78.

172. HKI. A Multi-faceted Approach to Improving Nutrition in Vietnam. 2018 [9/29/2021]. Available from: <https://helenkellerintl.org/our-stories/multi-faceted-approach-to-improving-nutrition-in-vietnam/>.

173. Bernet T, Kurbanalieva S, Pittore K, Zilly B, Luttikholt L, Eyhorn F, et al. Nutrition-sensitive agriculture interventions in mountain areas-Lessons learned from a 5-country project to upscale best practices. Mountain Research and Development. 2018;38(4):278-87. doi: <https://doi.org/10.1659/MRD-JOURNAL-D-18-00027.1>.

174. Leading Innovation and Impact in Nutrition Sensitive Agriculture in Africa. Helen Kellet International, n.d.

175. Behavior Change Strategy: Enhanced Homestead Food Production Project (Sengerema and Ukerewe Districts). Helen Keller International, n.d.

176. Keith N, Thiam K. Rapport sur la Recherche Formative: Project CHANGE. 2014.

177. HKI. Auto diagnostic communitaire des questions liees aux actions essentielles en nutrition, a l'hygiene & assainssement et a la production (vegetale & animale): Guide de l'animateur 2014.

178. Keith N. Résultats de la Recherche Formative Sur L’Hygiene et L’Entéropathie Environnementale Project CHANGE: Burkina Faso. Helen Keller International. 2014.

179. Talukder A, Haselow NJ, Osei AK, Villate E, Reario D, Kroeun H, et al. Homestead food production model contributes to improved household food security and nutrition status of young children and women in poor populations: lessons learned from scaling-up programs in Asia (Bangladesh, Cambodia, Nepal and Philippines). Field Actions Science Reports. 2010(Special Issue 1). <http://journals.openedition.org/factsreports/404>.

180. Gondwe T, Bussie M-D, Emmanuel Oladeji A, Mofu M, Numeri G. The relationship between training farmers in agronomic practices and diet diversification: a case study from an intervention under the Scaling Up Nutrition programme in Zambia. Agriculture & food security. 2017;6(1):72-. doi: <https://dx.doi.org/10.1186/s40066-017-0151-3>.

181. Kumar N, Nguyen PH, Harris J, Harvey D, Rawat R, Ruel MT. What it takes: evidence from a nutrition- and gender-sensitive agriculture intervention in rural Zambia. Journal of Development Effectiveness. 2018;10(3):341-72. doi: 10.1080/19439342.2018.1478874. PubMed PMID: WOS:000438404500005.

182. Rosenberg AM, Gelson T, Jody H, John AM, Marjolein M, Phuong HN, et al. Nutrition-sensitive agricultural interventions, agricultural diversity, food access and child dietary diversity: Evidence from rural Zambia. Food policy. 2018;80:10-23. doi: <https://dx.doi.org/10.1016/j.foodpol.2018.07.008>. PubMed PMID: IND606161131 Pagination: p. 10-23. Identifiers: Agricultural diversity.

183. Murendo C, Nhau B, Mazvimavi K, Khanye T, Gwara S. Nutrition education, farm production diversity, and commercialization on household and individual dietary diversity in Zimbabwe. Food Nutr Res. 2018;62. doi: 10.29219/fnr.v62.1276. PubMed PMID: 29849533.
