# Supplementary material for: Effectiveness of Elements of Social Behavior Change Activities in Nutrition-Sensitive Agriculture Programs: A Systematic Review
Source: Curr Dev Nutr. 2024 Jul 26;8(8):104420. doi: 10.1016/j.cdnut.2024.104420 (PMC11367542; doi:10.1016/j.cdnut.2024.104420)
Supplement: Multimedia component 7 [file mmc7.docx]

| **Appendix F: Projects excluded from the systematic review during later stages of review and abstraction** | |
| --- | --- |
| **Project Code (citation)** | **Reason for exclusion** |
| G04 (1) | No consumption focused SBC activities mentioned |
| K02 (2) | No consumption focused SBC activities mentioned |
| K07 (3) | No SBC activities described |
| SA03 (4) | No SBC activities described |
| Z01 (5) | No consumption focused SBC activities described |
| Be01 (6-8) | No consumption focused SBC activities described; only agriculture-related SBC activities |
| I03 (9) | No consumption focused SBC activities mentioned |
| K06 (10) | Not an intervention, secondary analysis of national data |
| K08 (11) | No consumption focused SBC activities mentioned |
| K07 (12) | No consumption focused SBC activities mentioned |
| R03 (13) | No consumption focused SBC activities described |
| Zi02 (14) | No consumption focused SBC activities described |

**References**

1. de Jager I, Abizari AR, Douma JC, Giller KE, Brouwer ID. Grain legume cultivation and children's dietary diversity in smallholder farming households in rural Ghana and Kenya. Food Secur. 2017;9:1053-71. Epub 2017/01/01. doi: 10.1007/s12571-017-0720-0. PubMed PMID: 32952744; PubMed Central PMCID: PMC7473086.

2. Butler LM, Bhandari S, Otieno P, Weiser SD, Cohen CR, Frongillo EA. Agricultural and Finance Intervention Increased Dietary Intake and Weight of Children Living in HIV-Affected Households in Western Kenya. Curr Dev Nutr. 2020;4(2):nzaa003. Epub 2020/01/31. doi: 10.1093/cdn/nzaa003. PubMed PMID: 31998859; PubMed Central PMCID: PMC6981349.

3. Walingo MK. Discriminant function analysis for tracing successful factors associated with livestock projects for nutrition improvement in western Kenya. East Afr J Public Health. 2009;6 Suppl(1):30-6. Epub 2010/01/21. doi: 10.4314/eajph.v6i3.45769. PubMed PMID: 20084983.

4. Ngema PZ, Sibanda M, Musemwa L. Household Food Security Status and Its Determinants in Maphumulo Local Municipality, South Africa. Sustainability. 2018;10(9):3307. <https://www.mdpi.com/2071-1050/10/9/3307>. PubMed PMID: doi:10.3390/su10093307.

5. Dumas SE, Lewis D, Travis AJ. Small-scale egg production centres increase children's egg consumption in rural Zambia. Matern Child Nutr. 2018;14 Suppl 3(Suppl 3):e12662. Epub 2018/10/18. doi: 10.1111/mcn.12662. PubMed PMID: 30332540; PubMed Central PMCID: PMC6865982.

6. Alaofe H, Burney J, Naylor R, Taren D. Solar-Powered Drip Irrigation Impacts on Crops Production Diversity and Dietary Diversity in Northern Benin. Food Nutr Bull. 2016;37(2):164-75. Epub 2016/03/25. doi: 10.1177/0379572116639710. PubMed PMID: 27009089.

7. Alaofe H, Burney J, Naylor R, Taren D. The impact of a Solar Market Garden programme on dietary diversity, women's nutritional status and micronutrient levels in Kalale district of northern Benin. Public Health Nutr. 2019;22(14):2670-81. Epub 2019/07/10. doi: 10.1017/S1368980019001599. PubMed PMID: 31280754; PubMed Central PMCID: PMC10260421.

8. Alaofe H, Zhu M, Burney J, Naylor R, Douglas T. Association Between Women's Empowerment and Maternal and Child Nutrition in Kalale District of Northern Benin. Food Nutr Bull. 2017;38(3):302-18. Epub 2017/04/27. doi: 10.1177/0379572117704318. PubMed PMID: 28443373.

9. Dubé L, McRae C, Wu Y-H, Ghosh S, Allen S, Ross D, et al. Impact of the eKutir ICT-enabled social enterprise and its distributed micro-entrepreneur strategy on fruit and vegetable consumption: A quasi-experimental study in rural and urban communities in Odisha, India. Food Policy. 2020;90:101787. doi: <https://doi.org/10.1016/j.foodpol.2019.101787>.

10. Jin M, Iannotti LL. Livestock production, animal source food intake, and young child growth: the role of gender for ensuring nutrition impacts. Soc Sci Med. 2014;105:16-21. Epub 2014/03/13. doi: 10.1016/j.socscimed.2014.01.001. PubMed PMID: 24606793.

11. Wyatt AJ, Yount KM, Null C, Ramakrishnan U, Webb Girard A. Dairy intensification, mothers and children: an exploration of infant and young child feeding practices among rural dairy farmers in Kenya. Matern Child Nutr. 2015;11(1):88-103. Epub 2013/08/15. doi: 10.1111/mcn.12074. PubMed PMID: 23941354; PubMed Central PMCID: PMC6860198.

12. Hoorweg J, Leegwater P, Veerman W. Nutrition in agricultural development: Intensive dairy farming by rural smallholders. Ecology of Food and Nutrition. 2000;39(6):395-416. doi: 10.1080/03670244.2000.9991627.

13. Del Prete D, Ghins L, Magrini E, Pauw K. Land consolidation, specialization and household diets: Evidence from Rwanda. Food Policy. 2019;83:139-49. doi: <https://doi.org/10.1016/j.foodpol.2018.12.007>.

14. Puett C, Salpeteur C, Lacroix E, Zimunya SD, Israel AD, Ait-Aissa M. Cost-effectiveness of community vegetable gardens for people living with HIV in Zimbabwe. Cost Eff Resour Alloc. 2014;12:11. Epub 2014/05/17. doi: 10.1186/1478-7547-12-11. PubMed PMID: 24834014; PubMed Central PMCID: PMC4022439.
